# A hybrid transmission model for *Plasmodium vivax* accounting for superinfection, immunity and the hypnozoite reservoir

**DOI:** 10.1007/s00285-024-02088-7

**Published:** 2024-05-21

**Authors:** Somya Mehra, Peter G. Taylor, James M. McCaw, Jennifer A. Flegg

**Affiliations:** 1 School of Mathematics and Statistics, The University of Melbourne, Parkville, Australia; 2 Centre for Epidemiology and Biostatistics, Melbourne School of Population and Global Health, The University of Melbourne, Parkville, Australia; 3 Peter Doherty Institute for Infection and Immunity, The Royal Melbourne Hospital and The University of Melbourne, Parkville, Australia

**Keywords:** Hypnozoite, Superinfection, Immunity, Vivax Malaria, Hybrid model, Infinite server queue, 92D30

## Abstract

Malaria is a vector-borne disease that exacts a grave toll in the Global South. The epidemiology of *Plasmodium vivax*, the most geographically expansive agent of human malaria, is characterised by the accrual of a reservoir of dormant parasites known as hypnozoites. Relapses, arising from hypnozoite activation events, comprise the majority of the blood-stage infection burden, with implications for the acquisition of immunity and the distribution of superinfection. Here, we construct a novel model for the transmission of *P. vivax* that concurrently accounts for the accrual of the hypnozoite reservoir, (blood-stage) superinfection and the acquisition of immunity. We begin by using an infinite-server queueing network model to characterise the within-host dynamics as a function of mosquito-to-human transmission intensity, extending our previous model to capture a discretised immunity level. To model transmission-blocking and antidisease immunity, we allow for geometric decay in the respective probabilities of successful human-to-mosquito transmission and symptomatic blood-stage infection as a function of this immunity level. Under a hybrid approximation—whereby probabilistic within-host distributions are cast as expected population-level proportions—we couple host and vector dynamics to recover a deterministic compartmental model in line with Ross-Macdonald theory. We then perform a steady-state analysis for this compartmental model, informed by the (analytic) distributions derived at the within-host level. To characterise transient dynamics, we derive a reduced system of integrodifferential equations, likewise informed by our within-host queueing network, allowing us to recover population-level distributions for various quantities of epidemiological interest. In capturing the interplay between hypnozoite accrual, superinfection and acquired immunity—and providing, to the best of our knowledge, the most complete population-level distributions for a range of epidemiological values—our model provides insights into important, but poorly understood, epidemiological features of *P. vivax*.

## Introduction

Despite decades of concerted control and elimination efforts, malaria persists as a grave cause of morbidity and mortality in the Global South, yielding an estimated 241 million cases and 627,000 deaths in 2020 alone (WHO 2021). The global malaria burden is largely driven by the parasites *Plasmodium falciparum* and *Plasmodium vivax*, with the transmission of both parasites mediated by *Anopheles* mosquito vectors. In light of its expansive geographical distribution, over three billion people are thought to be at risk of *P. vivax* infection (Battle 2019; Battle and Kevin Baird 2021).

The difficulty of eliminating *P. vivax*, in particular, is compounded by the fact that an infected individual can acquire a reservoir of dormant parasites, known as hypnozoites, hidden within their liver. The consequences of mosquito inoculation for *P. vivax* are two-fold: in addition to causing a primary (blood-stage) infection, an infective bite can lead to the establishment of an (undetectable) batch of hypnozoites in the liver. Following an indeterminate dormancy period, the activation of a single hypnozoite can give rise to (blood-stage) relapse. Long latency periods (in the order of 6 to 9 months) are characteristic of temperate strains, while short latency periods (typically spanning 3 to 6 weeks) tend to be more common in tropical settings (Battle 2014; White et al. 2016). Hypnozoite activation is believed to be a key driver of superinfection—which involves the co-circulation of multiple parasite broods in the bloodstream (Popovici 2018), and has potential consequences for the overall duration of blood-stage infection, ergo, opportunties for onward human-to-mosquito transmission.

The hypnozoite reservoir also has important implications for the acquisition of immunity (Mueller et al. 2013). Relapse-driven exposure to a large number of genetically-distinct clones in early childhood is believed to underpin the dynamics of acquired immunity to *Plasmodium vivax* (Koepfli et al. 2013). The mechanisms of immune protection for *P. vivax* are multi-faceted and highly complex, but are known to be stage-specific (see Antonelli et al. (2020) for a recent review). The majority of the immune response to *P. vivax* is believed to be targeted towards asexual blood-stage parasites. Here, we distinguish two manifestations of (asexual) blood-stage immunity:

- *Clinical/antidisease immunity* reduces the risk or severity of clinical symptoms during a blood-stage infection, with epidemiological data suggesting rapid acquisition (Mueller et al. 2013, 2015).
- *Antiparasite immunity* modulates the clearance of blood-stage infection (Deroost et al. 2016), and is typically modelled through accelerated parasite clearance rates and/or reduced parasite densities (Griffin 2010; White 2018a).

*Transmission-blocking immunity*, which modulates the infectivity of sexual blood-stage parasites (gametocytes) to mosquitoes, is also of note, with the mitigation of mosquito-stage development curtailing onward transmission (Gamage-Mendis et al. 1992; Mueller et al. 2013, 2015; de Jong et al. 2020). There is evidence to suggest that the gametocyte circulation is *not* hampered by clinical and antiparasite immunity (Joyner 2019).

Other forms of immunity are thought to be of comparatively limited consequence in *natural* transmission settings. Pre-erythrocytic immunity targets parasite forms (sporozoites) established directly through mosquito inoculation, prior to further liver-stage development of the parasite. Due to the potential for each sporozoite to develop into a hypnozoite, pre-erythrocytic immune protection has been hypothesised to substantially mitigate the relapse burden (Mueller et al. 2013; White et al. 2017); exposure to sporozoites in natural transmission settings, however, is believed to be insufficient to induce strong pre-erythrocytic immune protection (Mueller et al. 2013). Likewise, immune responses targeted towards liver-stage parasites, particularly hypnozoites, are poorly understood (Galinski and Barnwell 2008), but are generally considered to be relatively minor.

The joint dynamics of immunity and the hypnozoite reservoir are of epidemiological interest. The dichotomisation of both hypnozoite carriage and immune status (Kammanee et al. 2001; Ishikawa et al. 2003; Aguas et al. 2012; Roy et al. 2013) yields, in some senses, the simplest approach for characterising population-level transmission dynamics. Various models of immunity have been proposed under these dichotomised frameworks, ranging from imperviousness to reinfection (until immunity is lost) (Kammanee et al. 2001), to an elevated rate of recovery (antiparasite) (Ishikawa et al. 2003); reduced infectiousness to mosquitoes (transmission-blocking) (Roy et al. 2013); and necessarily asymptomatic blood-stage infection (clinical) (Aguas et al. 2012). A slightly extended model of transmission-blocking immunity, superinfection and hypnozoite accrual has been proposed by De Zoysa et al. (1991)—with the limitation that each individual can harbour up to two broods of hypnozoites and two overlapping relapses, and a discrete immunity level $\{0, 1, 2 \}$.

A more comprehensive characterisation of immunity and the hypnozoite reservoir has recently been performed by by White (2018a). Under a hypnozoite ‘batch’ model—whereby hypnozoites are stratified into ‘batches’, each characterised by a constant rate of relapse over the span of an exponentially-distributed lifetime, with an imposed upper bound *K* on concurrent batch carriage—White (2018a) account for the acquisition of both antidisease immunity (which reduces the probability of symptomatic infection) and antiparasite immunity (which results in an elevated rate of parasite clearance and a reduced probability of detection via light miscroscopy). In addition to being restricted to short-latency strains, the framework of White (2018a) ignores size variation in parasite inocula, as we noted in Mehra et al. (2022). Further, White (2018a) do not explicitly account for superinfection.

Here, we seek to characterise the interplay between hypnozoite accrual, superinfection and acquired immunity, for both short- and long-latency strains. In Mehra et al. (2023), we have recently proposed a transmission model for *P. vivax* that explicitly accounts for superinfection and (short-latency) hypnozoite accrual; both hypnozoite density and the multiplicity of blood-stage infection are specifically included in the state space. Under the deterministic model derived in Mehra et al. (2023), we can recover population-level distributions for various quantities of epidemiological interest without encountering the computational overheads that have curtailed previous efforts to model explicit hypnozoite densities (White 2018a). The conceptual underpinning of the model detailed in Mehra et al. (2023) is the within-host framework we introduced in Mehra et al. (2022), which captures hypnozoite and superinfection dynamics as a function of mosquito-to-human transmission intensity, or the force of reinfection (FORI), in a general transmission setting. In the present paper, we adopt an analogous mathematical construction to Mehra et al. (2023), extending our previous work to allow for long-latency (temperate) strains and the acquisition of transmission-blocking and antidisease immunity.

This paper is structured as follows. Section 2 focuses on the characterisation of within-host dynamics as a function of the FORI. We begin by extending the open network of queues introduced in Mehra et al. (2022), which describes the joint dynamics of superinfection and the hypnozoite reservoir, to include a discretised immunity level (Sect. 2.1); as observed in Mehra et al. (2022), this immunity level is governed by a shot noise process, akin to a previous model of antibody dynamics we introduced in Mehra et al. (2021). Rather than solving for the state probabilities based on the Kolmogorov forward differential equations for the queueing network (Sect. 2.2), we make use of the fact that the hypnozoites experience independent trajectories to derive a time dependent probability generating function (PGF) as in our previous work (Mehra et al. 2021, 2022) (Sect. 2.3). Specific models for antidisease and transmission-blocking immunity are proposed in Sects. 2.4 and 2.5 respectively. Section 3 concerns the construction of a novel hybrid transmission model (Nåsell 2013; Henry 2020), predicated on the coupling of expected host and vector dynamics. To recover the expected dynamics of the vector population, we consider the Kolmogorov forward differential equations for an underlying birth-death process (Sect. 3.1); while observing that the within-host probability mass function (PMF) can be regarded as the expected population-level frequency distribution of hypnozoite, superinfection and immunity states (Henry 2020). We then couple host and vector dynamics in an infinite compartment model (Sect. 3.2). Steady state analysis—including the identification of a bifurcation parameter governing the existence of endemic equilibria (Sect. 3.2.1) and a sensitivity analysis of endemic equilibrium solutions (Sect. 3.2.2)—is performed using the within-host distributions derived in Mehra et al. (2022). To characterise transient population-level dynamics, we adopt the approach detailed in Mehra et al. (2023) to derive a reduced system of IDEs—comprising an integral equation for the immunity-modulated probability of human-to-mosquito transmission (per bloodmeal), and a set of ordinary differential equations (ODEs) governing the number of (un)infected and latent mosquitoes over time (Sect. 3.3). As a function of the FORI derived under the reduced system of IDEs, we recover population-level distributions for various quantities of epidemiological interest—including the size of the (non)-latent hypnozoite reservoir; superinfection; the prevalence of clinical infection and the relative contribution of relapses to the infection burden—using the distributions derived in Mehra et al. (2022). We make some concluding remarks in Sect. 4.

## Within-host human dynamics: hypnozoite accrual, superinfection and immunity

We have previously derived the functional dependence within a given human host between the FORI, and the joint dynamics of blood-stage infection and the hypnozoite reservoir by constructing an open network of infinite server queues (Mehra et al. 2022). Here, we extend this model to allow for the acquisition of immunity. Following the approach detailed in Appendix C.3 of Mehra et al. (2022), we assume that the within-host acquisition of immunity is described by a generalised shot noise process such that:

- the clearance of each primary infection/relapse elicits a boost of unit magnitude;
- the lifetime of each boost is exponentially-distributed with mean 1/*w*; and
- the overall immunity level is given by the cumulative sum of boosts over the entirety of an individual’s infection history.

As noted in Mehra et al. (2022), this discretised model of immunity can be considered a variation of the antibody model proposed in Mehra et al. (2021), in which the clearance of each primary infection/relapse elicits a boost of random magnitude that is then subject to exponential decay at a fixed (deterministic) rate.

In Sect. 2.1 below, we propose an open network of infinite server queues to capture the within-host dynamics of superinfection, hypnozoite accrual and immune acquisition (Mehra et al. 2022). The Kolmogorov forward differential equations governing the time evolution of the joint PMF for the network are stated in Sect. 2.2. Instead of directly solving the Kolmogorov forward differential equations (which comprise an infinite-dimensional set of ODEs), we derive a joint PGF for the state of the network following a similar approach to Mehra et al. (2022) (Sect. 2.3). Specific models for transmission-blocking and antidisease immunity are detailed in Sects. 2.4 and 2.5 respectively. To elucidate the dynamics captured by our within-host model, we discuss an illustrative sample path in Sect. 2.6.

### An open network of infinite server queues

**Fig. 1:**
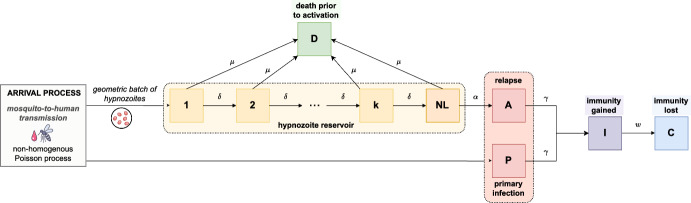
Schematic of open network of infinite server queues governing within-host hypnozoite and infection dynamics, allowing for the acquisition of immunity. Extended from Mehra et al. (2022) to include states related to immunity

To capture the within-host acquisition of immunity, we extend the open network of queues detailed in Mehra et al. (2022) to include an additional node *I*, such that the occupancy of queue *I* represents the immunity level. Specifically, we construct an open network of infinite server queues, labelled $1, \dots , k, NL, A, D, I, C, P$ (Fig. 1), where we define the compartments/nodes

- $i \in \{1, \dots , k \}$ to represent hypnozoites that are present in latency compartment *i* (that is, part of the hidden liver-stage reservoir but unable to activate);
- *NL* to represent non-latent hypnozoites (that is, part of the hidden liver-stage reservoir and able to activate);
- *A* to represent ongoing relapses from activated hypnozoites;
- *D* to represent hypnozoites that have died prior to activation;
- *P* to represent ongoing primary infections;
- *I* to represent cleared blood-stage infections (primary infections or relapses) that have given rise to an immunity increment of unit magnitude;
- *C* to represent the loss of immune memory.

As such, the state space for each hypnozoite is $S_h = \{ 1, \dots , k, NL, A, D, I, C\}$, while the state space for each primary infection is $S_p = \{ P, I, C \}$. A state vector specifies the number of hypnozoites in each of compartments $1, \dots , k, NL, A, D$, the number of primary stage infections in compartment *P*, together with the total amount of immunity gained from the clearance of blood stage immunity in compartment *I* and the total amount of immune memory that has been lost in compartment *C*.

Arrivals into the network, which represent infective bites, are modelled to follow a non-homogeneous Poisson process with rate $\lambda (t)$. The consequences of each infective bite are two-fold:

- a primary infection is immediately triggered, that is, a single “customer” enters queue *P*;
- a geometrically-distributed batch of hypnozoites (with mean size $\nu$) is established in the liver, entering latency compartment 1 in the case of long-latency strains ($k>0$); and the non-latent compartment *NL* in the case of short-latency strains ($k=0$).

Each hypnozoite/infection is assumed to flow independently through the network. Latent hypnozoites in the liver (that is, states $i \in \{1, \dots , k \}$) may either die at rate $\mu$, or shift to successive latency compartments at rate $\delta$. This equates to exponentially-distributed service times, with mean duration $1/(\delta + \mu )$, in each of queues $i \in \{1, \dots , k \}$. A departure from queue *i* is routed to either queue *D* (representing hypnozoite death) with probability $\mu /(\delta + \mu )$; or queue $(i+1)$ (representing progression to the next dormancy compartment) with probability $\delta /(\delta + \mu )$.

In contrast, non-latent hypnozoites (state *NL*) undergo death at rate $\mu$, and activation at rate $\alpha$. As such, service times in queue *NL* are modelled to be exponentially-distributed with mean $1/(\alpha + \mu )$, with departures routed either into queue *A* (in which case hypnozoite activation has triggered a relapse) with probability $\alpha /(\alpha + \mu )$, or queue *D* (in which case the hypnozoite has died prior to activation) with probability $\mu /(\alpha + \mu )$.

The clearance of each blood-stage infection is assumed to be independent, and modelled to occur at some constant rate $\gamma$, amounting to exponentially-distributed service times (with mean duration $1/\gamma$) in both queues *A* and *P* (representing relapses and primary infections respectively).

To capture the boosting of immunity with exposure, we assume that queue *I* receives all departures from queues *A* and *P* (corresponding to cleared blood-stage infections); that is, an immune boost of unit magnitude is acquired upon the clearance of each primary infection or relapse. To capture the waning of immune memory with time, we assume that each immune boost is retained for an exponentially-distributed period of time with mean 1/*w*—coinciding precisely with the service time in queue *I*. The occupancy of queue *I* therefore acts as a measure of within-host immunity. All departures from queue *I* are routed to queue *C*, where they remain indefinitely.

In a natural generalisation of this queueing network, the stratification of blood-stage infection and immunity into different compartments could allow us to capture additional stages of the parasite lifecycle and further biological realism.

#### Modelling correlates of immunity

We can formulate correlates of immune protection as time-dependent functionals of the state of the open network, with the host immunity level $N_I(t)$ mapped to the degree of immune protection at time *t*. To preserve the independence structure of the queueing network, however, these functionals cannot have any direct feedback into the within-host model. This limits the forms of immunity that are analytically tractable under our model.

A key assumption of the within-host model is that the arrival process is independent of the state of the network. As such, we cannot capture pre-erythrocytic immunity, which modulates the probability of successful mosquito-to-human transmission, whereby the arrival process, comprising mosquito bites, would depend on the occupancy $N_I(t)$ of node *I*. The assumption of independence between service rates within each node and the state of queueing network is equally important. We are thus unable to account for the potential modulation of (blood-stage) parasite clearance rates—or equivalently, the service rate for nodes *A* and *P*—as a function of the host immunity level $N_I(t)$, that is, the occupancy of node *I*. We could, however, introduce *deterministic* time variation in the rate of clearance of blood-stage infection $\gamma$ to model age-related physiological factors.

Immune correlates that are amenable under our within-host framework include:

- The probability of exhibiting clinical symptoms or high-density parasitemia, as a manifestation of antidisease immunity (Sect. 2.4).
- The probability of human-to-mosquito transmission when an uninfected mosquito takes a bloodmeal from a blood-stage infected human host, as a measure of transmission-blocking immunity (Sect. 2.5).

There is evidence to suggest that these forms of immunity are acquired on different time scales. Transmission-blocking immune memory, for instance, is believed to be relatively short-lived, with boosting driven largely by successive blood-stage infections in intervals of $<4$ months (Gamage-Mendis et al. 1992); antidisease immunity, in contrast, is believed to be more robust and longer-lived (Mueller et al. 2013). By augmenting the rate of decay of the probability of symptomatic blood-stage infection (antidisease) as a function of $N_I(t)$, relative to the probability of human-to-mosquito transmission (transmission-blocking), we allow for strong antidisease protection to develop more rapidly than transmission-blocking protection, and be maintained at lower transmission intensities. Before discussing these immune correlates, however, we derive an analytic expression for the distribution of the state of the queueing network at time *t* (Mehra et al. 2022).

### Kolmogorov forward differential equations

Denote by $N_s(t)$ the number of hypnozoites/infections in each state $s \in S:= S_h \cup S_p$ at time *t* and set

$$\begin{aligned} H_{i_1, \dots , i_k, i_{NL}, j, k}(t) = P&(N_1(t) = i_1, \dots , N_k(t) = i_k, N_{NL}(t) = i_{NL},\\&\,\, N_A(t) + N_P(t) = j, N_I(t) = k). \end{aligned}$$

Then by the Kolmogorov forward differential equations, the time evolution of the state probabilities $H_{i_1, \dots , i_k, i_{NL}, j, k}(t)$ is governed by the countable system of ODEs

1 $$\begin{aligned}&\frac{d H_{i_1, \dots , i_k, i_{NL},j,k}}{dt} = \underbrace{\lambda (t) \bigg [ -H_{i_1, \dots , i_k, i_{NL},j,k}(t) + \sum ^{i_1}_{\ell =0} \frac{1}{\nu + 1} \Big ( \frac{\nu }{ \nu + 1} \Big )^{i_1 - \ell } H_{\ell , \dots , i_k, i_{NL},j-1,k}(t) \bigg ]}_{\text {reinfection (geometric batch of hypnozoites + primary infection triggered)}} \nonumber \\&\quad + \underbrace{\mu \bigg [ - \Bigg ( \sum ^k_{\ell =1} i_\ell + i_{NL} \Bigg ) H_{i_1, \dots , i_k, i_{NL},j,k}(t) + \sum ^k_{\ell =1} (i_\ell +1) H_{i_1, \dots , i_{\ell - 1}, i_\ell + 1, i_{\ell +1}, \dots i_k, i_{NL},j,k}(t) + (i_{NL}+1) H_{i_1, \dots , i_k, i_{NL} + 1,j,k}(t) \bigg ]}_{\text {death of a hypnozoite in the liver (latent or non-latent) prior to activation}} \nonumber \\&\quad + \underbrace{\delta \bigg [ - \sum ^k_{\ell =1} i_\ell H_{i_1, \dots , i_k, i_{NL},j,k}(t) + \sum ^{k-1}_{\ell =1} (i_\ell + 1) H_{i_1, \dots , i_{\ell - 1}, i_\ell + 1, i_{\ell +1} -1, \dots i_k, i_{NL},j,k}(t) + (i_{k}+1) H_{i_1, \dots i_k + 1, i_{NL} - 1,j,k}(t) \bigg ]}_{\text {progression of a latent hypnozoite to the next latency compartment}} \nonumber \\&\quad + \underbrace{\alpha \Big [ - i_{NL} H_{i_1, \dots , i_k, i_{NL},j,k}(t) + (i_{NL}+1) H_{i_1, \dots , i_k, i_{NL}+1,j-1,k}(t) \Big ]}_{\text {activation of a non-latent hypnozoite, triggering a relapse}} \nonumber \\&\quad + \underbrace{\gamma \Big [ -j H_{i_1, \dots , i_k, i_{NL},j,k}(t) + (j+1) H_{i_1, \dots , i_k, i_{NL},j+1,k-1}(t) \Big ]}_{\text {clearance of blood-stage infection + gain of immunity increment}} \nonumber \\&\quad + \underbrace{w \Big [ - k H_{i_1, \dots , i_k, i_{NL},j,k}(t) + (k+1) H_{i_1, \dots , i_k, i_{NL},j,k+1}(t) \Big ]}_{\text {waning of immune memory}}. \end{aligned}$$

Consider a human population of fixed size $P_H$, with each individual taken to be immune- and infection-naive at time zero. In the absence of demography (that is, human births and deaths), we can re-interpret the within-host PMF $H_{i_1, \dots , i_k, i_{NL}, j, k}(t)$ as the *expected* proportion of humans with $i_m$ hypnozoites in state $m \in \{1, \dots , k, NL\}$; a blood-stage infection comprising *j* parasite broods and having an immunity level *k*. Equation (1) can therefore be viewed as governing the *expected* proportion of humans in each hypnozoite/infection/immunity state (Henry 2020). In Sect. 3.2, we will draw on Eq. (1) to construct a hybrid transmission model, comprising a countably infinite system of ODEs. Our aim in the present section, however, is to characterise the within-host PMF $H_{i_1, \dots , i_k, i_{NL}, j, k}(t)$. While the infinite-dimensional system of ODEs given by Eq. (1) is difficult to solve, we can readily derive the joint PGF governing the state of the network following similar reasoning to Mehra et al. (2022).

### The joint PGF for the state of the network

Rather than solving Eq. (1) to yield the probability mass function (PMF) for the state of the queue directly, we derive a joint PGF

$$\begin{aligned}&{{\,\mathrm{\mathbbm {E}}\,}}\Big [ \prod _{s \in S} z_s^{N_s(t)} \Big ]\\&\quad = \sum ^\infty _{i_1=0} \dots \sum ^\infty _{i_k=0} \sum ^\infty _{i_{NL}=0} \sum ^\infty _{j=0} \sum ^\infty _{k=0} z_1^{i_1} \cdot \dots \cdot z_k^{i_k} \cdot z_{NL}^{i_{NL}} \cdot z_A^{j} z_I^{k} \cdot H_{i_1, \dots , i_k, i_{NL}, j, k}(t) \end{aligned}$$

for the state of the network from first principles, using an argument which is an extension of that in Mehra et al. (2022). Like the PMF, the PGF can be viewed as an alternative way of characterising the time-dependent distribution of the network. A description of the properties of PGFs can be found in Chapter XI of Feller (1968). Under the assumption of geometrically-distributed batch arrivals, we can invert the marginal PGF to recover PMFs for quantities of epidemiological interest, as discussed in Mehra et al. (2022).

Treating the dynamics of each hypnozoite/infection to be independent (Harrison and Lemoine 1981), we begin by characterising the probability mass function for a single hypnozoite/primary infection that enters the network at time zero. Here, we extend the activation-clearance model proposed by White et al. (2014)—and discussed in detail in Mehra et al. (2020)—to allow for the clearance of blood-stage infection (which was also examined in Mehra et al. (2022)) and the gain/loss of immunity (as introduced in the present manuscript). To characterise hypnozoite dynamics, we consider the flow of an arrival into either queue *NL* (for short-latency strains) or queue 1 (for long-latency strains) through the queueing network shown in Fig. 1. Similarly, the dynamics of each primary infection are described by the flow of an arrival into queue *P* through the network.

Denote by $p_{h,s}(t)$ the probability that a hypnozoite established at time zero is in compartment $s \in S_h$ at time *t*. By the Kolmogorov forward differential equations, it follows that

2 $$\begin{aligned} \frac{dp_{h, 1}}{dt}&= -(\delta + \mu ) p_{h,1}(t) \end{aligned}$$

3 $$\begin{aligned} \frac{dp_{h, \ell }}{dt}&= -(\delta + \mu ) p_{h,\ell }(t) + \delta p_{h, \ell - 1}(t) \text { for } \ell \in \{2, \dots , k \} \end{aligned}$$

4 $$\begin{aligned} \frac{dp_{h, NL}}{dt}&= -(\alpha + \mu ) p_{h,NL}(t) + \delta p_{h,k}(t) \end{aligned}$$

5 $$\begin{aligned} \frac{dp_{h, A}}{dt}&= -\gamma p_{h, A}(t) + \alpha p_{h,NL}(t) \end{aligned}$$

6 $$\begin{aligned} \frac{dp_{h, I}}{dt}&= -w p_{h,I}(t) + \gamma p_{h,A}(t) \end{aligned}$$

7 $$\begin{aligned} \frac{dp_{h, C}}{dt}&= w p_{h,I}(t) \end{aligned}$$

8 $$\begin{aligned} \frac{dp_{h, D}}{dt}&= \mu \Bigg ( \sum ^k_{i=1} p_{h,i}(t) + p_{h, NL}(t) \Bigg ) \end{aligned}$$

with the initial condition

9 $$\begin{aligned} p_h(0)&= {\left\{ \begin{array}{ll} (p_{h,1}(0), \dots , p_{h,k}(0), p_{h,NL}(0), p_{h,A}(0), p_{h,C}(0), p_{h,D}(0))\quad \text {if } k>0\\ (p_{h,NL}(0), p_{h,A}(0), p_{h,C}(0), p_{h,D}(0))\quad \text {if } k =0 \end{array}\right. }\nonumber \\&= {\left\{ \begin{array}{ll} (1, 0, \dots , 0, 0, 0, 0, 0)\quad \text {if } k > 0\\ (1, 0, 0, 0)\quad \text {if } k = 0 \end{array}\right. } \end{aligned}$$

Following similar reasoning to Mehra et al. (2020, 2022), we can solve the system of ODEs given by Eqs. (2)–(6) analytically; solutions are given in Eqs. (33) to (36) in Appendix A. Since we do not require the distribution of dead hypnozoites or cleared infections over time for further analysis presented, we do not provide solutions to the ODEs (7) and (8).

Likewise, we can characterise the probabilistic time course for each primary infection. Denote by $p_{p,s}(t)$ the probability that a primary infection triggered at time zero is in state $s \in S_p$ at time *t*. We can solve the Kolmogorov forward equations

$$\begin{aligned} \frac{dp_{p,P}}{dt} = -\gamma p_{p,P}(t) \qquad \frac{dp_{p,I}}{dt} = -w p_{p,I}(t) + \gamma p_{p,P}(t) \qquad \frac{dp_{p,C}}{dt}&= w p_{p,I}(t) \end{aligned}$$

with initial condition

$$\begin{aligned} \textbf{p}_p(0) = (p_{p,P}(0), p_{p,I}(0), p_{p,C}(0)) = (1, 0,0) \end{aligned}$$

to yield

10 $$\begin{aligned}&p_{p, P} = e^{-\gamma t} \qquad \qquad p_{p, I} = \frac{\gamma }{\gamma - w} \Big ( e^{-w t} - e^{-\gamma t} \Big ) \qquad \qquad \nonumber \\&p_{p, C}(t) = 1 - p_{p,P}(t) - p_{p, I}(t). \end{aligned}$$

Embedding these state probabilities in an epidemiological framework, as elucidated in Mehra et al. (2022), the joint PGF for

$$\begin{aligned} \textbf{N}(t) = (N_1(t), \dots , N_k(t), N_{NL}(t), N_A(t), N_D(t), N_I(t), N_C(t), N_P(t)) \end{aligned}$$

is given by

11 $$\begin{aligned}&G(t, z_1, \dots , z_k, z_{NL}, z_A, z_D, z_C, z_I, z_{P}) := {{\,\mathrm{\mathbbm {E}}\,}}\Big [ \prod _{s \in S} z_s^{N_s(t)} \Big ] \nonumber \\&\quad = \exp \bigg \{ - \int ^t_0 \lambda (\tau ) \bigg [ 1 - \frac{\sum _{s \in S_p} z_s \cdot p_{p, s}(t - \tau )}{1 + \nu \big ( 1- \sum _{s \in S_h} z_s \cdot p_{h,s}(t-\tau ) \big )} \bigg ] d \tau \bigg \}. \end{aligned}$$

In Mehra et al. (2022), we recovered analytic expressions for the distributions of several biologically-relevant quantities—encompassing the size of the (non)-latent hypnozoite reservoir; the number of parasite broods co-circulating in the bloodstream; the relative contribution of relapses to the infection burden and the cumulative number of recurrences (that is, primary infections and relapses) experienced over time—using the joint PGF given by Eq. (11). Formulae relevant to the present manuscript are recapitulated in Appendix B.

### Antidisease immunity

While the number of broods co-circulating in the bloodstream at time *t* is given by the total occupancy of nodes *A* and *P*, that is, $N_A(t) + N_P(t)$, a large proportion of *P. vivax* infections in endemic settings are asymptomatic, with implications for treatment and elimination strategies (Almeida 2018; Tadesse 2018; Ferreira 2022). The relative burden of (a)symptomatic blood-stage infection, which is a function of antidisease immunity, is therefore of epidemiological interest.

Conditional on the presence of blood-stage infection, we assume that the probability of an individual exhibiting clinical symptoms decreases by a factor of $p_c$ for each increment of immunity they harbour. As such, the probability of an individual with state $\textbf{N}(t)$ exhibiting clinical symptoms is given by

$$\begin{aligned} p_\text {clin}(t) = p_{c}^{N_I(t)} \cdot \mathbbm {1}_{\{ N_A(t) + N_P(t) > 0 \}}. \end{aligned}$$

Accounting for stochasticity in within-host hypnozoite and infection dynamics, the probability of an individual exhibiting clinical symptoms at time *t* can be written

12 $$\begin{aligned} p_\text {clin}(t)&:= \sum ^\infty _{i_1=0} \dots \sum ^\infty _{i_k=0} \sum ^\infty _{i_{NL}=0} \sum ^\infty _{j=1} \sum ^\infty _{k=0} p_{c}^k H_{i_1, \dots , i_k, i_{NL}, j, k}(t) \nonumber \\&= {{\,\mathrm{\mathbbm {E}}\,}}\Big [ p_{c}^{N_I(t)} \big | N_A(t) + N_P(t)> 0 \Big ] \cdot P \big ( N_A(t) + N_P(t) > 0 \big ) \nonumber \\&= {{\,\mathrm{\mathbbm {E}}\,}}\Big [ p_{c}^{N_I(t)} \Big ] - {{\,\mathrm{\mathbbm {E}}\,}}\Big [ p_{c}^{N_I(t)} \big | N_A(t) + N_P(t) = 0 \Big ] \cdot P \big ( N_A(t) + N_P(t) = 0 \big ) \end{aligned}$$

using the law of total expectation. Setting $z_i = 1$ for $i \in S {\setminus } I$ in Eq. (11) to recover the marginal PGF for $N_I(t)$, we can write

13 $$\begin{aligned}&{{\,\mathrm{\mathbbm {E}}\,}}\Big [ p_{c}^{N_I(t)} \Big ] \nonumber \\&\quad = G(t, z_1=1, \dots , z_k=1, z_{NL}=1, z_A=1, z_D=1, z_C=1, z_I=p_{c}, z_{P}=1). \end{aligned}$$

By Xekalaki (1987), we can recover the unnormalised PGF for $N_I(t)$, conditional on the absence of blood-stage infection (that is, $N_A(t) + N_P(t) = 0$), by firstly setting $z_i = 1$ for $i \in S {\setminus } \{ A, P, I \}$ in Eq. (11) (to recover the joint PGF for $(N_A(t), N_P(t), N_I(t))$, and then setting $z_A = z_P = 0$ (to exclusively consider the case $N_A(t) + N_P(t) = 0$), yielding the expression

14 $$\begin{aligned}&{{\,\mathrm{\mathbbm {E}}\,}}\Big [ p_{c}^{N_I(t)} \big | N_A(t) + N_P(t) = 0 \Big ] \cdot P \big ( N_A(t) + N_P(t) = 0 \big ) \nonumber \\&\quad = G(t, z_1=1, \dots , z_k=1, z_{NL}=1, z_A=0, z_D=1, z_C=1, z_I=p_{c}, z_{P}=0). \end{aligned}$$

Substituting Eqs. (13) and (14) into Eq. (12), we recover an expression for $p_\text {clin}(t)$ as a function of the FORI $\lambda (\tau )$ in the interval $\tau \in [0, t)$:

15 $$\begin{aligned} p_\text {clin}(t)&= \exp \bigg \{ - \int ^t_0 \lambda (\tau ) I_M(\tau ) \bigg [ 1 - \frac{1 - (1-p_c) p_{{p}, I}(t - \tau )}{1 + \nu (1-p_c) p_{{h}, I}(t-\tau )} \bigg ] d \tau \bigg \} \nonumber \\&\quad - \exp \bigg \{ - \int ^t_0 \lambda (\tau ) \bigg [ 1 - \frac{1 - (1-p_c) \cdot p_{p, I}(t-\tau ) - p_{p, A}(t-\tau ) }{1 + \nu (1-p_c) p_{h, I}(t-\tau ) + \nu p_{h, A}(t-\tau )} d \tau \bigg \} \end{aligned}$$

where we have used the joint PGF given by Eq. (11). In a similar vein, we can introduce analogous models linking the probabilities of (sub)microscopic parasitemia and detectability (through light microscopy versus rapid diagnostic tests versus qPCR assays) to the immunity level $N_I(t)$.

### Transmission-blocking immunity

Here, we propose a model for transmission-blocking immunity by introducing a functional dependence between the probability of successful human-to-mosquito transmission and the immune status of a blood-stage infected individual.

For an immune-naive, blood-stage infected individual, we set the probability of successful human-to-mosquito transmission to be $p_0$. We further assume that the probability of successful human-to-mosquito transmission is reduced by a factor of $p_{tb} \in [0, 1]$ for each increment of immunity. Suppose a mosquito takes a bloodmeal from a human with state $\textbf{N}(t)$ at time *t*. Conditional on the state of a human host $\textbf{N}(t)$, we thus define the probability of successful human-to-mosquito transmission $p_{h \rightarrow m}(t)$ to be

$$\begin{aligned} p_{h \rightarrow m}(t) = p_0 \cdot p_{tb}^{N_I(t)} \cdot \mathbbm {1}_{\{ N_A(t) + N_P(t) > 0 \}}. \end{aligned}$$

Under our stochastic epidemiological framework, following similar reasoning to Sect. 2.4, we can recover the probability of successful human-to-mosquito transmission when a mosquito bites an individual at time *t* as a function of the FORI $\lambda (\tau )$ in the interval $\tau \in [0, t)$

16 $$\begin{aligned}&p_{h \rightarrow m}(t) := p_0 \sum ^\infty _{i_1=0} \dots \sum ^\infty _{i_k=0} \sum ^\infty _{i_{NL}=0} \sum ^\infty _{j=1} \sum ^\infty _{k=0} p_{tb}^k H_{i_1, \dots , i_k, i_{NL}, j, k} \nonumber \\&\quad = p_0 \Big ( {{\,\mathrm{\mathbbm {E}}\,}}\Big [ p_{tb}^{N_I(t)} \Big ] - {{\,\mathrm{\mathbbm {E}}\,}}\Big [ p_{tb}^{N_I(t)} \big | N_A(t) + N_P(t) = 0 \Big ] \cdot P \big ( N_A(t) + N_P(t) = 0 \big ) \Big ) \nonumber \\&\quad = p_0 \Big [ G(t, z_1=1, \dots , z_k=1, z_{NL}=1, z_A=1, z_D=1, z_C=1, z_I=p_{c}, z_{P}=1)\nonumber \\&\qquad \qquad - G(t, z_1=1, \dots , z_k=1, z_{NL}=1, z_A=0, z_D=1, z_C=1, z_I=p_{c}, z_{P}=0) \Big ] \nonumber \\&\quad = p_0 \Bigg [ \exp \bigg \{ - \int ^t_0 \lambda (\tau ) \bigg [ 1 - \frac{1 - (1-p_{tb}) p_{{p}, I}(t - \tau )}{1 + \nu (1-p_{tb}) p_{{h}, I}(t-\tau )} \bigg ] d \tau \bigg \} \nonumber \\&\qquad \qquad - \exp \bigg \{ - \int ^t_0 \lambda (\tau ) \bigg [ 1 - \frac{1 - (1-p_{tb}) \cdot p_{p, I}(t-\tau ) - p_{p, A}(t-\tau ) }{1 + \nu (1-p_{tb}) p_{h, I}(t-\tau ) + \nu p_{h, A}(t-\tau )} d \tau \bigg \} \Bigg ]. \end{aligned}$$

Note that Eq. (16) accounts for both the acquisition of immunity and the probability of blood-stage infection.

The quantity $p_{h \rightarrow m}(t)$ is of particular importance since it underpins the coupling between host and vector dynamics; the time evolution of the expected number of infected mosquitoes (and consequently, the FORI) is dependent only on $p_{h \rightarrow m}(t)$ and several (known) transmission parameters (see Sect. 3.1). Equation (16) will be of particular use in Sect. 3.3, where we construct a reduced hybrid transmission model.

**Fig. 2:**
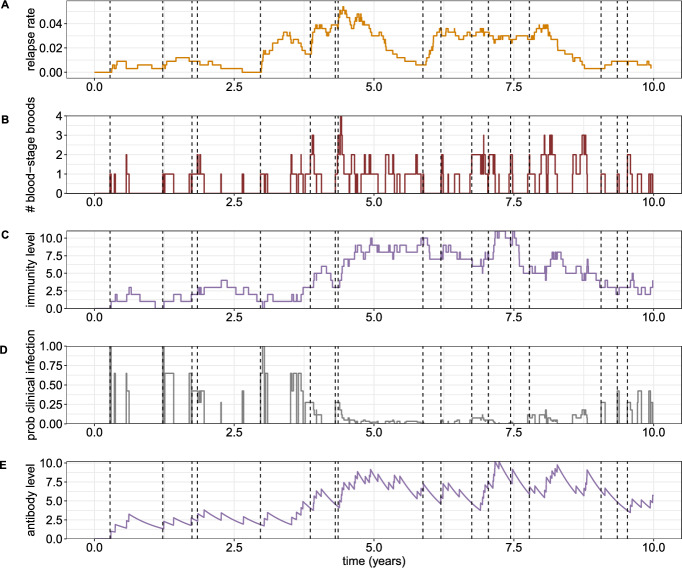
Sample path, obtained through direct stochastic simulation, for an individual subject to a constant FORI ($\lambda = 2$ year^-1^) over a period of 10 years. At time zero, the individual is immune-naive, and harbours neither liver- or blood-stage infection. Each infective bite (indicated with a dashed vertical line) triggers a primary infection, in addition to establishing a geometrically-distributed batch of hypnozoites in the liver, with mean size $\nu = 6.4$, as per White et al. (2016). We consider long-latency hypnozoites, with $k=2$ and $\delta = 1/100$ day^-1^. Values for the hypnozoite activation $\alpha = 1/334$ day^-1^ and death $\mu = 1/442$ day^-1^ rates are drawn from White et al. (2014). Blood-stage infections (primary and relapse) are assumed to be cleared at constant rate $\gamma = 1/24$ day^-1^, as per estimates from White (2018b). Under the discretised model of immunity, the lifetime of each immunity boost is assumed to be exponentially-distributed with mean duration $1/w = 250$ days, with the probability of clinical symptoms (conditional on the presence of blood-stage infection) assumed to decrease by a factor of $p_c=0.65$ for each increment of immunity. **A** The rate of relapse $\alpha N_{NL}(t)$. **B** The number of parasite broods $N_A(t) + N_P(t)$ co-circulating in the bloodstream. **C** The discretised immunity level $N_I(t)$. **D** The probability of clinical infection $p_c^{N_I(t)} \mathbbm {1}_{\{ N_A(t) + N_P(t) > 0 \}}$. **E** The antibody level, as per the model introduced in Mehra et al. (2021), whereby the clearance of each blood-stage infection is associated with a unit boost of immunity that then decays exponentially (deterministically) with rate 1/250 day^-1^

### An illustrative sample path

A simulated sample path illustrating temporal variation in the relapse rate, superinfection status (that is, the number of co-circulating parasite broods in the bloodstream) and immunity level, is shown in Fig. 2, as an extension of previous simulations presented in Mehra et al. (2021, 2022). We assume that the individual is both immune- and infection-naive at time zero, and is subject to a constant FORI $\lambda =2$ year^-1^. Each bite is modelled to establish a geometrically-distributed batch of hypnozoites of mean size $\nu =6.4$ (White et al. 2016), with activation rate $\alpha =1/334$ day^-1^ (White et al. 2014), death rate $\mu =1/442$ day^-1^ (White et al. 2014) and a long-latency characteristic ($k=2$, $\delta =1/100$ day^-1^). Each blood-stage infection is modelled to undergo clearance at rate $\gamma =1/24$ day^-1^ (White 2018b), and confer an immunity increment of mean duration $1/w=250$ days with the probability of clinical infection decaying geometrically by a factor of $p_c=0.65$ per immune increment.

Over the course of 10 years, the simulated individual is subject to 17 infective mosquito bites (shown with vertical dashed lines). The temporal variation in the relapse rate (Fig. 2A), which is proportional to the size of the non-latent hypnozoite reservoir, arises from the interplay between hypnozoite replenishment (through mosquito inoculation) and clearance (through either activation or death). Blood-stage infections include both primary infections (triggered immediately upon mosquito inoculation) and relapses (triggered by hypnozoite activation), with temporally proximate reinfection and/or hypnozoite activation events yielding multiple blood-stage infections (Fig. 2B). The discretised immunity level (shown in Fig. 2C) likewise fluctuates, with the clearance of each blood-stage infection eliciting a unit boost that is retained for an exponentially-distributed period of time. The probability of clinical infection (which serves as a correlate of antidisease immunity) decays geometrically with the discretised immunity level (Fig. 2D). For comparison, in Fig. 2E, we illustrate the simplest case of our previous (continuous) models of antibody dynamics (Mehra et al. 2021), whereby the clearance of each blood-stage infection is associated with a unit boost of immunity that decays exponentially (deterministically). Both the discrete (Fig. 2C) and continuous (Fig. 2E) models of immunity yield qualitatively similar results in this case.

## Hybrid transmission models: coupling expected host and vector dynamics

We now construct hybrid transmission models (Nåsell 2013; Henry 2020) to couple host and vector dynamics. To the best of our knowledge, these models are novel in structure, in that hypnozoite densities, the multiplicity of blood-stage infection and an immunity level are explicitly included in the state space.

We restrict our attention to a homogeneously mixing population of humans and mosquitoes. While the size of the human population $P_H$ is taken to be fixed (with no age structure), we allow for time-variation in the size of the mosquito population (e.g. due to climactic variation or the implementation of vector-based control). Vector dynamics are detailed in Sect. 3.1.

In Mehra et al. (2023), for a simpler model structure—accounting only for superinfection and short-latency hypnozoite accrual—we began by constructing a Markov population process (with countably many types) to couple host and vector dynamics. Using the work of Barbour and Luczak (2012), we then recovered a deterministic (infinite) compartmental model as a functional law of large numbers; that is, we showed that the sample paths of the Markov process converged to a deterministic sample path in the infinite population size limit, when the number of mosquitoes per human was held fixed. We noted there, however, that an identical deterministic compartmental model could be recovered under a “hybrid approximation”, whereby host and vector dynamics are coupled through expected values, as per the construction of Nåsell (2013); Henry (2020). This hybrid construction is the focus of Sect. 3.2; by regarding the within-host PMF as the expected population-level frequency distribution (Henry 2020), we recover a compartmental model (comprising an infinite-dimensional system of ODEs) that can be viewed as natural extension of the Ross-Macdonald model to allow for superinfection, hypnozoite accrual and immune acquisition. We characterise endemic equilibria for this compartmental model by drawing on results derived at the within-host level (Sect. 3.2.1), before performing a sensitivity analysis (Sect. 3.2.2).

To characterise the transient dynamics of the hybridised system, we adopt the strategy we introduced in Mehra et al. (2023). Specifically, we collapse the infinite-dimensional compartmental model into a reduced system of IDEs—with a set of ODEs governing the time evolution of the number of (un)infected and latent mosquitoes over time; and an integral equation governing the (transmission-blocking) immunity-modulated probability of successful human-to-mosquito tranmsmission (Sect. 3.3). Based on the time evolution of the number of infected mosquitoes under the reduced system of IDEs, we can recover population-level distributions for several quantities of epidemiological interest using our derived within-host distributions.

### Vector dynamics: birth, death and infectivity

Here, we characterise the dynamics of the vector population. We assume mosquito-dynamics are described by a Markovian birth-death process, whereby:

- Mosquito births follow a time-dependent rate $\omega (t)$ (reflecting, for instance, climactic variation)
- Mosquito lifetimes are exponentially-distributed with mean duration 1/*g*;
- Each mosquito bites humans (within a population of fixed size $P_H$) at a potentially time-varying rate $\beta (t)$ (reflecting, for instance, the relaxation/intensification of vector-based control measures);
- Following successful human-to-mosquito transmission (due to a bloodmeal from a blood-stage infected human), a mosquito undergoes sporogony (that is, the development of ingested malaria parasites into sporozoites that can be transmitted onwards to other humans) at rate $\eta$;
- After sporogony has occured, a mosquito remains infective to humans until death.

A mosquito that is undergoing sporogony following successful human-to-mosquito transmission is hereafter considered to be latent.

Denote by $I_M(t)$, $L_M(t)$, $U_M(t)$ the expected number of infected, latent and uninfected mosquitoes respectively at time *t*. By the Kolmogorov forward differential equations for the Markovian birth-death process governing the vector population (see Appendix C for details), we obtain the system of coupled ODEs

17 $$\begin{aligned} \frac{dI_M}{dt}&= \eta L_M(t) - g I_M(t) \end{aligned}$$

18 $$\begin{aligned} \frac{dL_M}{dt}&= \beta (t) p_{h \rightarrow m}(t) U_M(t) - (g + \eta ) L_M(t) \end{aligned}$$

19 $$\begin{aligned} \frac{dU_M}{dt}&= \omega (t) \big [ I_M(t) + L_M(t) + U_M(t) \big ] - \big [ \beta (t) p_{h \rightarrow m}(t) + g \big ] U_M(t) \end{aligned}$$

governing the time evolution of $I_M(t)$, $L_M(t)$, $U_M(t)$ as a function of the probability of human-to-mosquito transmission $p_{h \rightarrow m}(t)$ per bloodmeal.

### A countable system of ODEs

Under a hybrid approximation, we seek to couple expected host and vector dynamics (Nåsell 2013; Henry 2020). Here, we recall two key observations:

- As we noted in Sect. 2.2, for a human population of fixed size $P_H$, the system of ODEs given by Eq. (1) governs the *expected* proportion of humans in each hypnozoite/infection/immunity state as a function of the FORI (Henry 2020).
- The time evolution of the *expected* number of (un)infected mosquitoes in the population is governed by Eqs. (17) and (19) conditional on the probability of successful human-to-mosquito transmission per bloodmeal.

To construct a hybrid transmission model, as per the approach of Nåsell (2013), it remains to characterise the FORI, and the probability of human-to-mosquito transmission.

We assume that mosquito-to-human transmission occurs with fixed probability $p_{m \rightarrow h}$ when a human is bitten by an infected mosquito. As a function of the number of infected $I_M(t)$ mosquitoes over time, the FORI can therefore be written

20 $$\begin{aligned} \lambda (t) = \frac{\beta (t) p_{m \rightarrow h} I_M(t)}{P_H}. \end{aligned}$$

Likewise, as a function of the proportion of humans $H_{i_1, \dots , i_k, i_{NL}, j, k}(t)$ with $i_{\ell }$ hypnozoites in state $\ell \in \{1, \dots , k, NL \}$; *j* co-circulating broods in the bloodstream; and immunity level *k* at time *t*, the probability of successful human-to-mosquito transmission $p_{h \rightarrow m}(t)$ can be written

$$\begin{aligned} p_{h \rightarrow m}(t) = p_0 \sum ^\infty _{i_1=0} \dots \sum ^\infty _{i_k=0} \sum ^\infty _{i_{NL}=0} \sum ^\infty _{j=1} \sum ^\infty _{k=0} p_{tb}^k H_{i_1, \dots , i_k, i_{NL},j,k}(t) \end{aligned}$$

as per the model of transmission-blocking immunity detailed in Sect. 2.5.

**Fig. 3:**
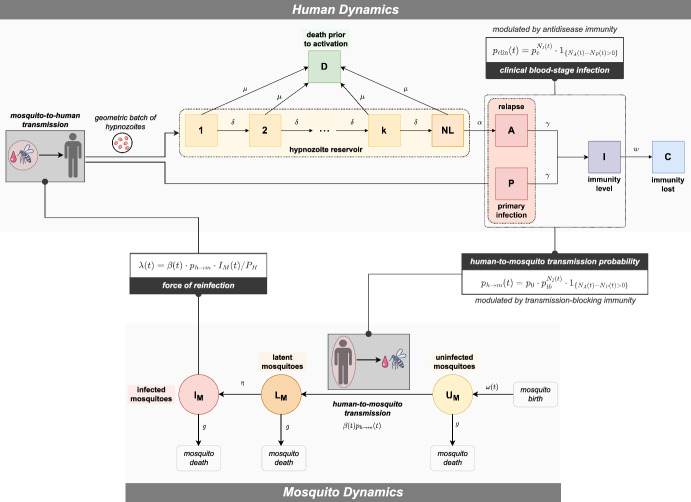
Schematic of hybrid transmission model structure, predicated on the coupling of expected host and vector dynamics (Nåsell 2013; Henry 2020). Here, the probabilistic distribution of the open network of infinite server queues governing within-host dynamics (Sect. 2.1) is re-interpreted as the expected proportion of humans in each hypnzoite/superinfection/immunity state. The coupling of host and vector dynamics is predicated on the force of reinfection $\lambda (t)$ (Eq. (20)), which is a function of the number of infected mosquitoes at time *t*; and the probability of successful human-to-mosquito transmission $p_{h \rightarrow m}(t)$ per bloodmeal (Eq. (16)), which is modulated both by the prevalence of blood-stage infection and the distribution of immunity in the human population at time *t*

Then following a similar approach to Nåsell (2013), we recover the countable system of ODEs

21 $$\begin{aligned}&\frac{d H_{i_1, \dots , i_k, i_{NL},j,k}}{dt} = \beta (t) p_{m \rightarrow h} \frac{I_M(t)}{P_H}\bigg [ -H_{i_1, \dots , i_k, i_{NL},j,k}(t) + \sum ^{i_1}_{\ell =0} \frac{1}{\nu + 1} \Big ( \frac{\nu }{ \nu + 1} \Big )^{i_1 - \ell } H_{\ell , \dots , i_k, i_{NL},j-1,k}(t) \bigg ] \nonumber \\&\quad + \mu \bigg [ - \bigg ( \sum ^k_{\ell =1} i_\ell + i_{NL} \bigg ) H_{i_1, \dots , i_k, i_{NL},j,k}(t) + \sum ^k_{i=1} (i_\ell +1) H_{i_1, \dots , i_{\ell - 1}, i_\ell + 1, i_{\ell +1}, \dots i_k, i_{NL},j,k}(t) \nonumber \\&\quad \qquad + (i_{NL}+1) H_{i_1, \dots , i_k, i_{NL} + 1,j,k}(t) \bigg ] \nonumber \\&\quad + \delta \bigg [ - \sum ^k_{\ell =1} i_\ell H_{i_1, \dots , i_k, i_{NL},j,k}(t) + \sum ^{k-1}_{i=1} (i_\ell + 1) H_{i_1, \dots , i_{\ell - 1}, i_\ell + 1, i_{\ell +1} -1, \dots i_k, i_{NL},j,k}(t) \nonumber \\&\quad \qquad + (i_{k}+1) H_{i_1, \dots i_k + 1, i_{NL} - 1,j,k}(t) \bigg ] \nonumber \\&\quad + \alpha \Big [ - i_{NL} H_{i_1, \dots , i_k, i_{NL},j,k}(t) + (i_{NL}+1) H_{i_1, \dots , i_k, i_{NL}+1,j-1,k}(t) \Big ] \nonumber \\&\quad + \gamma \Big [ -j H_{i_1, \dots , i_k, i_{NL},j,k}(t) + (j+1) H_{i_1, \dots , i_k, i_{NL},j+1,k-1}(t) \Big ] \nonumber \\&\quad + w \Big [ - k H_{i_1, \dots , i_k, i_{NL},j,k}(t) + (k+1) H_{i_1, \dots , i_k, i_{NL},j,k+1}(t) \Big ] \end{aligned}$$

22 $$\begin{aligned}&\frac{dI_M}{dt} = \eta L_M(t) - gI_M(t) \end{aligned}$$

23 $$\begin{aligned}&\frac{dL_M}{dt} = \beta (t) p_0 \sum ^\infty _{i_1=0} \dots \sum ^\infty _{i_k=0} \sum ^\infty _{i_{NL}=0} \sum ^\infty _{j=1} \sum ^\infty _{k=0} p_{tb}^k H_{i_1, \dots , i_k, i_{NL},j,k}(t) U_M(t) - (g + \eta ) L_M(t) \end{aligned}$$

24 $$\begin{aligned}&\frac{dU_M}{dt} = \omega (t) ( I_M(t) + L_M(t) + U_M(t)) \nonumber \\&\quad \qquad - \bigg ( g + \beta (t) p_0 \sum ^\infty _{i_1=0} \dots \sum ^\infty _{i_k=0} \sum ^\infty _{i_{NL}=0} \sum ^\infty _{j=1} \sum ^\infty _{k=0} p_{tb}^k H_{i_1, \dots , i_k, i_{NL},j,k}(t) \bigg ) U_M(t), \end{aligned}$$

where we have used Eqs. (1) and (17)–(20). A schematic of this model is provided in Fig. 3.

Equations (21) and (24) could also have been written down as a deterministic comparmental model, representing a natural extension of the Ross-Macdonald framework to allow for hypnozoite accrual, superinfection and transmission-blocking immunity. In the special case of short-latency strains ($k=0$) with no transmission-blocking immunity ($p_{tb}=1$); no time variation in the vector parameters $\beta (t)=\beta$ and $\omega (t)=\omega$; and instantaneous sporogony (that is, in the limit $\eta \rightarrow \infty$), our model structure collapses down to the simpler model developed in Mehra et al. (2023). We can also view Eqs. (21) and (24) as an extension of the transmission model proposed by White et al. (2014) to allow for long-latency hypnozoites, immunity and explicit superinfection dynamics (as opposed to the approximation of superinfection through an appropriate recovery rate). A summary of model parameters, and their respective interpretations, is provided in Table 1.

**Table 1:** Summary of model parameters

Parameter	Interpretation	Value	Source
$\alpha$	Hypnozoite activation rate	1/334 day^-1^	White et al. (2014)
$\mu$	Hypnozoite death rate	1/442 day^-1^	White et al. (2014)
$\delta$	Rate of progression through hypnozoite latency compartments	1/100 day^-1^	Assumed
*k*	Number of hypnozoite latency compartments	0, 1, 2	Assumed
$\nu$	Mean number of hypnozoites established per bite	6.4	White et al. (2016)
$\gamma$	Rate at which each blood-stage infection is cleared	1/24 day^-1^	White (2018b)
*w*	Rate at which each immune increment/boost is lost	1/250 day^-1^	Assumed
$p_{c}$	Factor by which the probability of clinical/symptomatic blood-stage infection decreases per unit level of immunity	Various	Assumed
$p_0$	Probability of human-to-mosquito transmission when a mosquito bites a blood-stage infected, immune-naive human	0.25, 0.65	Assumed
$p_{tb}$	Factor by which the probability of human-to-mosquito transmission decreases per unit level of immunity when a mosquito bites a blood-stage infected human	0.9	Assumed
$p_{h \rightarrow m}(t)$	Probability of human-to-mosquito transmission when an uninfected mosquito takes a bloodmeal at time *t*	Calculated	Eq. (16)
$p_{m \rightarrow h}$	Probability of mosquito-to-human transmission when an infected mosquito bites a human	0.25	White (2018a)
$\beta (t)$	Bite rate per mosquito	0.21 day^-1^	Garrett-Jones (1964)
*g*	Mosquito death rate	0.1 day^-1^	Gething et al. (2011)
$\omega (t)$	Mosquito birth rate at time *t*	Various	Assumed
$1/\eta$	Mean duration of sporogony	12 days	Gething et al. (2011)
$\frac{P_M}{P_H}$	Ratio of mosquito and human population size assuming $\omega (t)=g$	1.2	Assumed
$\lambda (t)$	Force of reinfection (FORI) at time *t*	Calculated	Eq. (20)

Source values for parameters have been provided where possible, but we have assumed some values otherwise

#### The stationary solution

Here, we seek to characterise steady state solutions to the system of ODEs given by Eqs. (21)–(24). As such, we restrict ourselves to a setting where:

- The bite rate per mosquito $\beta (t) = \beta$ remains constant over time; and
- The mosquito population size $I_M(t) + L_M(t) + U_M(t)= P_M$ is fixed, that is, the birth rate $\omega (t) = g$ exactly balances the (constant) death rate.

Denote by $H^*_{i_1, \dots , i_k, i_{NL}, j,k}$, $U^*_M$, $L^*_M$ $I^*_M$ the stationary solution to the system of IDEs given by Eqs. (21) to (24), recovered by setting all time derivatives to zero.

We focus on the quantities $H^*_{i_1, \dots , i_k, i_{NL}, j,k}$ and $I^*_M$ since the overarching effect of sporogony is to introduce a scaling factor ${\eta }/(g+\eta )$ in the fraction of mosquitoes that, in the event of successful mosquito-to-human transmission, survive latency to transition from an uninfected to infected state. Setting the time derivative in Eq. (22) to zero and using the assumption that the mosquito population size is fixed, we can formulate the number of latent $L_M^*$ and uninfected $U_M^*$ mosquitoes at steady state as functions of $I_M^*$ and $P_M$:

$$\begin{aligned} L_M^* = \frac{g}{\eta } I_M^* \qquad U_M^* = P_M - \Big ( 1 + \frac{g}{\eta } \Big ) I_M^*. \end{aligned}$$

We observe that the disease (and immunity) free equilibrium $H^*_{0, \dots , 0, 0, 0, 0}=1, I^*_M=0$ always exists. Here, we seek to characterise the existence of endemic equilibrium solutions.

We begin by setting the time derivatives in Eqs. (22) and (24) to zero to yield

25 $$\begin{aligned} \sum ^\infty _{i_1=0} \dots \sum ^\infty _{i_k=0} \sum ^\infty _{i_{NL}=0} \sum ^\infty _{j=1} \sum ^\infty _{k=0} p_{tb}^k H^*_{i_1, \dots , i_k, i_{NL},j,k} = \frac{g I_M^*}{\beta p_0 \big ( \frac{P_M}{1+g/\eta } -I^*_M \big )}. \end{aligned}$$

We then note that Eq. (21) is precisely the set of Kolmogorov forward differential equations for the open network of infinite server queues introduced in Sect. 2. The PGF for the stationary limiting distribution of this queueing network, given a constant FORI $\lambda (t) = \beta p_{m \rightarrow h} I^*_M/P_H$, can be recovered by taking the limit $t \rightarrow \infty$ in Eq. (11). Therefore, using Eq. (16)—which we derived from Eq. (11) in Sect. 2.5—it must be the case that

26 $$\begin{aligned}&\sum ^\infty _{i_1=0} \dots \sum ^\infty _{i_k=0} \sum ^\infty _{i_{NL}=0} \sum ^\infty _{j=1} \sum ^\infty _{k=0} p_{tb}^k H^*_{i_1, \dots , i_k, i_{NL},j,k} \nonumber \\&\quad = p_0 \Bigg ( \exp \bigg \{ - \frac{\beta p_{m \rightarrow h} I^*_M}{P_H} \int ^\infty _0 \bigg [ 1 - \frac{1 - (1-p_{tb}) p_{{p}, I}(\tau )}{1 + \nu (1-p_{tb}) p_{{h}, I}(\tau )} \bigg ] d \tau \bigg \} \nonumber \\&\qquad - \exp \bigg \{ - \frac{\beta p_{m \rightarrow h} I^*_M}{P_H} \int ^\infty _0 \bigg [ 1 - \frac{1 - (1-p_{tb}) \cdot p_{p, I}(\tau ) - p_{p, A}(\tau ) }{1 + \nu (1-p_{tb}) p_{h, I}(\tau ) + \nu p_{h, A}(\tau )} d \tau \bigg \} \Bigg ). \end{aligned}$$

Using a simple geometric argument (Appendix D), we can show that Eqs. (25) and (26) have at most one non-zero intersection (corresponding to an endemic equilibrium solution), and that this intersection exists if and only if

27 $$\begin{aligned} R_0^2&:= \frac{\beta ^2 p_0 p_{m \rightarrow h} P_M}{g (1 + g/\eta ) P_H}\nonumber \\&\quad \int ^\infty _0 \bigg [ \frac{1 - (1-p_{tb}) p_{{p}, I}(\tau )}{1 + \nu (1-p_{tb}) p_{{h}, I}(\tau )} - \frac{1 - (1-p_{tb}) \cdot p_{p, I}(\tau ) - p_{p, A}(\tau ) }{1 + \nu (1-p_{tb}) p_{h, I}(\tau ) + \nu p_{h, A}(\tau )} \bigg ] d \tau > 1. \end{aligned}$$

The bifurcation parameter $R_0^2$ is amenable to interpretation. Denote by $T_{h \rightarrow m}$ the total expected duration of transmissible blood-stage infection following a given mosquito bite, allowing for the contribution of both relapse and primary infection, and the progressive acquisition of transmission-blocking immunity attributable to the bite itself. In Appendix D.1, we show that

$$\begin{aligned} T_{h \rightarrow m} = p_0 \int ^\infty _0 \bigg [ \frac{1 - (1-p_{tb}) p_{p, I}(\tau )}{1 + \nu (1-p_{tb}) p_{h, I}(\tau )} - \frac{1 - (1-p_{tb}) \cdot p_{p, I}(\tau ) - p_{p, A}(\tau ) }{1 + \nu (1-p_{tb}) p_{h, I}(\tau ) + \nu p_{h, A}(\tau )} \bigg ] d \tau . \end{aligned}$$

Therefore, using Eq. (27), we can write the quantity $R_0^2$ in the formas a product of interpretable components. The quantity $R_0$ is thus a reproduction number (Diekmann et al. 1990; Mehra et al. 2023).

Assuming that $R_0 >1$ (Eq. (27)), an endemic equilibrium solution necessarily exists. As a function of the FORI $\lambda ^* =\beta p_{m \rightarrow h} I^*_M/P_H$ at the endemic equilibrium, which, in turn, is a function of the non-trivial solution $I_M^* \in (0, P_M]$ to Eqs. (25) and (26), we can recover population-level distributions for various quantities of epidemiological interest using the stationary limiting PGF recovered by setting $\lambda (t) = \lambda ^*$ for all $t \ge 0$ and taking the limit $t \rightarrow \infty$ in Eq. (11). Relevant formulae (based on the derivations presented in Mehra et al. (2022)) are provided in Appendix B.

#### Sensitivity analysis for endemic equilibrium solutions

We now perform a sensitivity analysis for the endemic equilibrium solutions. In Sect. 3.2.2.1, we examine endemic equilibrium solutions in the absence of immunity. Endemic equilibria, allowing for transmission-blocking and antidisease immunity, are detailed in Sect. 3.2.2.2.

**3.2.2.1 Short-latency vs long-latency strains in the absence of transmission-blocking immunity** We begin by examining steady state solutions for both short-latency ($k=0$) and long-latency ($k>0$) strains in the absence of transmission-blocking immunity ($p_{tb}=1$).

**Fig. 4:**
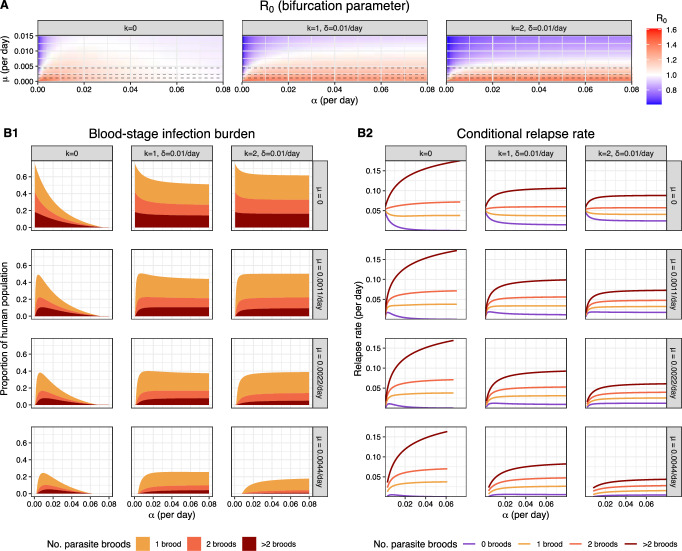
Three-way sensitivity analysis (with respect to $\mu$, $\alpha$ and *k*) for steady state solutions solutions in the absence of transmission-blocking immunity ($p_{tb}=1$) for both short-latency ($k=0$) and long-latency ($k>0$) strains. We fix $p_0=0.25$ and parameters $\gamma , \nu , g = \omega (t), \beta , p_{m \rightarrow h}, P_M/P_H$ as per Table 1. **A** Sensitivity analysis for $R_0$ (Eq. (27)). Here, we consider $\mu \in [0, 0.015)$ day^-1^ and $\alpha \in [0, 0.08)$ day^-1^, each in increments of 0.00055 day^-1^; and $k=0,1,2$, while fixing $\delta =1/100$ day^-1^. Values of $\mu$ considered in **B** are indicated with dashed horizontal lines. **B** Sensitivity analysis for (**B1**) the number of co-circulating parasite broods (Eqs. (39)–(41)); and (**B2**) the relapse rate conditional on superinfection status (Eqs. (44)–(47)) at the endemic equilibrium solution. The prevalence of blood-stage infection and the number of infected mosquitoes $I_M^*$ at the endemic equilibrium are given by the non-trivial solution to Eqs. (26) and (25) (which exists, and is unique, iff $R_0>1$); endemic equilibrium solutions for quantities of epidemiological interest are recovered as functions of $I_M^*$. Here, we consider $\mu \in \{0, 0.0011, 0.0022, 0.0044\}$ day^-1^; $\alpha \in [0, 0.08)$ day^-1^ in increments of 0.00055 day^-1^; and $k=0,1,2$, while fixing $\delta =1/100$ day^-1^

Figure 4A depicts a sensitivity analysis for the bifurcation parameter $R_0$ (Eq. (27)) as a function of the hypnozoite activation $\alpha$, death $\mu$ and latency *k* parameters, with the other parameter values given in Table 1. Recall that an endemic equilibrium exists, and is unique, if $R_0 > 1$; if $R_0<1$, only the disease-free equilibrium exists. The bifurcation boundary $R_0 = 1$ for parameters $(\alpha , \mu )$ is shown in white in Fig. 4A. In the absence of hypnozoite accrual ($\nu = 0$), no endemic equilibria exist for the set of transmission parameters considered here; the existence of endemic equilibrium solutions is therefore contingent on the relapse burden. The interplay between hypnozoite activation $\alpha$ and death $\mu$ rates governs the expected number of relapses per bite ${\nu } \alpha /(\alpha + \mu )$ (White et al. 2014). As such, when the hypnozoite activation rate $\alpha$ is low relative to the hypnozoite death rate $\mu$, there are insufficient relapses to sustain transmission and no endemic equilibrium solution exists, that is, $R_0 < 1$ (Fig. 4A).

In the case of short-latency strains ($k=0$), we further observe that excessively high activation rates $\alpha$ preclude the existence of endemic equilibrium solutions (that is, yield $R_0 < 1$) (Fig. 4A); similar observations have been posited by White et al. (2016) and Anwar et al. (2022), albeit in the absence of superinfection. Without an enforced dormancy period, an elevated activation rate $\alpha$ gives rise to a high risk of relapse immediately after each infective bite. The rapid depletion of the hypnozoite reservoir following each bite—driven by temporally proximate hypnozoite activation events—leads to a divergence in relapse risk conditional on status of blood-stage (super)infection (Fig. 4B2). To justify why a high relapse rate for (blood-stage) superinfected individuals is a weaker driver of onward transmission than a high relapse rate for blood-stage uninfected individuals, we observe that the expected time to clearance for *j* parasite broods is $\gamma (1 + 1/2 + \dots + 1/j)$; as such, an additional relapse for an individual with *m* pre-existing broods in their bloodstream increases the expected time to (blood-stage) recovery by an increment of $\gamma /(m+1)$. We deduce that the stratification of relapse risk, conditional on the status of blood-stage infection, is driven by the time to the most recent infective bite in the case of fast-activating short-latency hypnozoites: while recently-inoculated individuals experience a high burden of both liver- and blood-stage infection, there is a limited burden of liver- and blood-stage infection *between* successive mosquito bites, yielding a population-level reduction in the overall burden of blood-stage infection (Fig. 4B1). Hence, for elevated activation rates $\alpha$, there is a limited window of time following each infective bite for which an individual remains blood-stage infected, and therefore infective to mosquitoes; if the activation rate $\alpha$ is sufficiently high, then these windows of human-to-mosquito infectivity may be insufficient to sustain transmission in the steady state, in which case $R_0<1$ and no endemic equilibrium solution exists (Fig. 4A).

For long-latency strains ($k>0$), stochasticity in the enforced dormancy period prevents excessive overlap between hypnozoite activation events arising from the same bite, thereby reducing the sensitivity of the endemic equilibrium burden of blood-stage infection to elevated hypnozoite activation rates $\alpha$ (Fig. 4B1). Decreasing the variance of the dormancy period $k/\delta ^2$, whilst fixing the expected duration $k/\delta$, would presumably increase the sensitivity of endemic equilibria to the hypnozoite activation rate $\alpha$, since hypnozoites would be more likely to emerge from dormancy at similar times. We observe that the assumption of independent dormancy, introduced in Mehra et al. (2020), underpins this observation for long-latency strains; the collective dormancy assumption of White et al. (2014)—whereby synchronicity in the latency phase means that hypnozoites established through the same infective bite emerge collectively from dormancy—leads to greater sensitivity of endemic equilibrium solutions to elevated hypnozoite activation rates $\alpha$. Indeed, under a ‘binary’ hypnozoite model predicated implicitly on the assumption of collective dormancy, White et al. (2016) predict stronger constraints on the hypnozoite activation rate $\alpha$ than we predict in Fig. 4 under the assumption of independent dormancy.

**Fig. 5:**
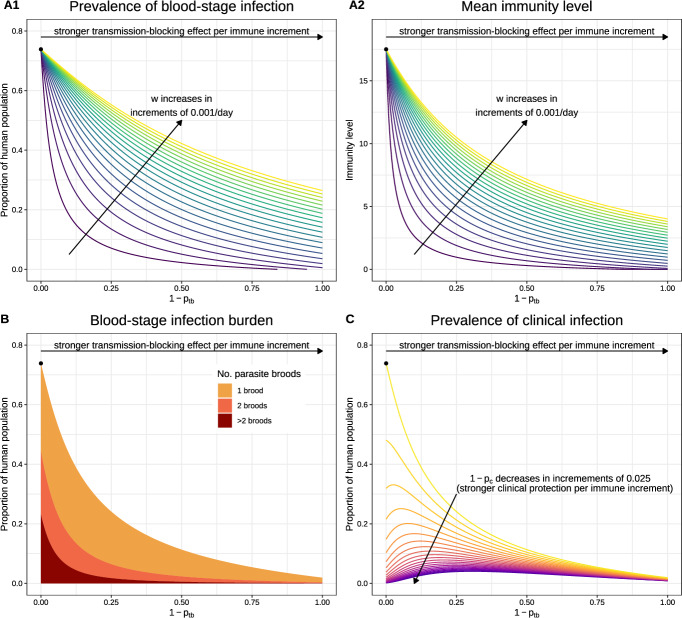
Endemic equilibrium solutions for for short-latency strains ($k=0$) allowing for transmission-blocking immunity ($0 < (1 - p_{tb}) \le 1$). We fix $p_{0}=0.65$ and parameters $\alpha$, $\mu$, $\gamma$, $\nu$, $g=\omega (t)$, $\beta$, $p_{m \rightarrow h}$, $P_M/P_H$ as per Table 1. The probability of human-to-mosquito transmission (per bloodmeal) and the number of infected mosquitoes $I_M^*$ at the endemic equilibrium are given by the non-trivial solution to Eqs. (26) and (25) (which exists, and is unique, iff $R_0>1$). Endemic equilibrium solutions for quantities of epidemiological interest are recovered as functions of $I_M^*$. **A** Two-way sensitivity analysis (with respect to $(1-p_{tb})$ and *w*) for **A1** prevalence of blood-stage infection (Eq. (39)) and **A2** the mean immunity level (Eq. (48)) at the endemic equilibrium. Here, we consider $w \in [0.001, 0.02)$ day^-1^ in increments of 0.001 day^-1^ and $(1-p_{tb}) \in [0, 1)$ in increments of 0.005. **B** One-way sensitivity analysis (with respect to $p_{tb}$) for the number of co-circulating parasite broods in the bloodstream (Eqs. (39)–(41)), with $w=1/250$ day^-1^ fixed and $(1-p_{tb}) \in [0,1)$ in increments of 0.005. **C** Two-way sensitivity analysis (with respect to $p_{tb}$ and $p_c$) for the prevalence of clinical infection (Eq. (15)) at the endemic equilibrium, where we consider $(1-p_{tb}) \in [0, 1)$ in increments of 0.005 and $p_c \in [0.5, 1)$ in increments of 0.025.

Elevated hypnozoite activation rates $\alpha$, however, give rise to a stratification of relapse risk by superinfection status, even in the case of long-latency strains ($k>0$) (Fig. 4B2). In the absence of hypnozoite death (that is, $\mu =0$), the burden of blood-stage infection at the endemic equilibrium is maximised for low hypnozoite activation rates $\alpha$ (Fig. 4B1), which yield broad temporal relapse distributions for each infective bite, and a population-level relapse risk that does not vary strongly by superinfection status (Fig. 4B2). For non-zero death rates $\mu >0$, however, hypnozoite death during the enforced dormancy period—during which activation is prohibited—serves as a key constraint. As such, elevated activation rates $\alpha$ (up to a point) yield an increasing burden of blood-stage infection for long-latency strains ($k>0$, Fig. 4B1), even as the risk of relapse stratified by superinfection status becomes more unbalanced and proportionately higher for individuals with pre-existing blood-stage infections (Fig. 4B2).

**3.2.2.2 Short-latency strains with transmission-blocking immunity** We now perform a sensitivity analysis for endemic equilibrium solutions allowing for the acquisition of transmission-blocking and antidisease immunity (Fig. 5). Here, we restrict ourselves to short-latency strains ($k=0$). For a fixed set of hypnozoite activation and death rates $(\alpha , \mu )$, long-latency strains ($k>0$) yield similar qualitative patterns as a function of the immunity parameters *w*, $p_{tb}$ and $p_c$. In the absence of transmission-blocking and clinical immunity (that is, $p_{tb}=p_c=1$), we revert to the setting examined in Sect. 3.2.2.1; we highlight this case with closed circles in Fig. 5.

A two-way sensitivity analysis, with respect to parameters *w* and $(1-p_{tb})$, is shown in Fig. 5A, with remaining parameter values detailed in Table 1. Recall that the probability of human-to-mosquito transmission (when an uninfected mosquito takes a bloodmeal from a blood-stage infected human) decays geometrically, with factor $p_{tb}$, as a function of an individual’s immunity level (Sect. 2.5); since protection rises as $p_{tb} \rightarrow 0$, we think of $(1-p_{tb})$ as a transmission-blocking protection parameter. In contrast, the parameter 1/*w* governs the time scale for which immunity is retained, with the limiting case $w = 0$ corresponding to a scenario where immunity is never lost. The endemic equilibrium prevalence of blood-stage infection (Fig. 5A1) decreases both as:

- immunity becomes longer-lived (that is, $w \rightarrow 0$), whereby a larger subset of an individual’s infection history is expected to contribute to their current immunity level; and
- the protective effect associated with each cleared blood-stage infection is augmented (that is, $(1-p_{tb}) \rightarrow 1$).

Mitigation of the blood-stage infection burden in light of transmission-blocking immunity, however, necessarily limits exposure; reductions in the prevalence of blood-stage infection are therefore accompanied by reductions in the population-level distribution of immunity. The mean immunity level at the endemic equilibrium therefore *decreases*, even as the rate of immune decay *w* decreases, and immunity accrues over a larger time scale (Fig. 5A2). Likewise, augmenting the transmission-blocking effect of each immunity increment $(1-p_{tb})$—whereby the probability of human-to-mosquito is suppressed strongly, even at low immunity levels—leads to a reduction in the mean immunity level at the endemic equilibrium (Fig. 5A2).

In particular, we see a substantially reduced burden of (blood-stage) superinfection at the endemic equilibrium as the transmission-blocking effect of each immune increment $(1-p_{tb})$ increases (Fig. 5B). The suppression of superinfection explains the stronger decay in the mean immunity level (Fig. 5A2), relative to the prevalence of blood-stage infection (Fig. 5A1), as the transmission-blocking protection parameter $(1-p_{tb})$ is strengthened: since the clearance of each primary infection and relapse yields an immunity boost, irrespective of temporal overlap with other blood-stage infections, superinfection is an important driver of acquired immunity.

We also observe a trade-off between transmission-blocking and antidisease immunity. The mitigation of transmission as the transmission-blocking protection parameter $(1-p_{tb})$ is augmented leads to a lower population-level distribution of immunity at the endemic equilibrium (Fig. 5A2). If the distribution of immunity at the endemic equilibrium is sufficiently reduced, then an increasingly strong transmission-blocking effect per immune increment $(1-p_{tb})$ can give rise to an increasing prevalence of clinical infection at the endemic equilibrium (Fig. 5C), even as the burden of blood-stage infection continues to decline (Fig. 5B).

### A reduced system of IDEs to study transient behaviour

The hybrid transmission model given by Eqs. (21)–(24) yields population-level dynamics of superinfection, the hypnozoite reservoir and acquired immunity. However, the countable system of ODEs given by Eqs. (21)–(24) is not necessarily readily amenable to numerical solution; truncating the system at reasonable endpoints could yield thousands of coupled ODEs, since the size of the hypnozoite reservoir (and, by extension, the immunity level) could reasonably be expected to range up to 30 in moderate to high transmission settings (see Figure 4 of White et al. (2014) and Figure 6 of Anwar et al. (2022)).

Here, we propose a reduced system of integrodifferential equations (IDEs) to couple host and vector dynamics, following the approach detailed in Mehra et al. (2023). In particular, we observe that:

- The dependence of the human population on vector dynamics can be distilled into the FORI, which is proportional to the number of infected mosquitoes in the population $I_M(t)$ (see Eq. (20)).
- The dependence of the vector population on the state of the human population can be distilled into the probability of successful human-to-mosquito transmission $p_{h \rightarrow m}(t)$ when an uninfected mosquito bites *any* human in the population; note that the quantity $p_{h \rightarrow m}(t)$ accounts for *both* the prevalence of blood-stage infection and the distribution of (transmission-blocking) immunity within the human population.

At time $t=0$, we make the assumption that each individual in the human population (of fixed size $P_H$) has immunity level zero; carries no hypnozoites; and harbours no ongoing blood-stage infections, whereby $p_{h \rightarrow m}(0) =0$. As such, we consider the introduction of a number of infected mosquitoes into an otherwise infection- and immune-naive human population.

Recall from Sect. 2.5 that

$$\begin{aligned} p_{h \rightarrow m}(t) = p_0 \sum ^\infty _{i_1=0} \dots \sum ^\infty _{i_k=0} \sum ^\infty _{i_{NL}=0} \sum ^\infty _{j=1} \sum ^\infty _{k=0} p_{tb}^k H_{i_1, \dots , i_k, i_{NL}, j, k}(t) \end{aligned}$$

where the population-level proportions $H_{i_1, \dots , i_k, i_{NL}, j, k}(t)$ satisfy Eq. (21), which constitutes the Kolmogorov forward differential equation for the queueing network analysed in Sect. 2. Under the initial condition

28 $$\begin{aligned} H_{0, \dots , 0, 0, 0, 0}(0) = 1, \quad H_{i_1, \dots , i_k, i_{NL}, j, k}(0) = 0 \text { for all } i_1 + \dots + i_k + i_{NL} + j + k > 0, \end{aligned}$$

given a time-varying function $\lambda (t):= \beta (t) p_{m \rightarrow h} I_M(t)/P_H$, the integral expression given by Eq. (16) constitutes the time-dependent state distribution that satisfies the Kolmogorov forward differential Eqs. (21). Rather than monitoring the time evolution of the population-level proportions $H_{i_1, \dots , i_k, i_{NL}, j, k}(t)$, we can directly substitute the weighted sum $p_{m \rightarrow h}(t)$ into Eqs. (23) and (24) to collapse the countable system of ODEs given by Eqs. (21) to (24) into a reduced system of IDEs.

We can motivate this construction under a hybrid approximation as follows. As a function of the FORI, the probability of successful human-to-mosquito transmission $p_{h \rightarrow m}(t)$ is governed by Eq. (16). Likewise, as a function of the probability of successful human-to-mosquito transmission $p_{h \rightarrow m}(t)$ per bloodmeal, the time evolution of the expected number of infected mosquitoes over time $I_M(t)$ is governed by the coupled system of ODEs given by Eqs. (22) to (24), which also captures the time evolution of the expected number of latent $L_M(t)$ and uninfected $U_M(t)$ mosquitoes over time.

Coupling expected host and vector dynamics under a hybrid approximation thus yields the system of IDEs

29 $$\begin{aligned} \frac{dI_M}{dt}&= \eta L_M(t) - gI_M(t) \end{aligned}$$

30 $$\begin{aligned} \frac{dL_M}{dt}&= \beta (t) p_{h \rightarrow m}(t) U_M(t) - (g + \eta ) L_M(t) \end{aligned}$$

31 $$\begin{aligned} \frac{dU_M}{dt}&= \omega (t) \big [ I_M(t) + L_M(t) + U_M(t) \big ] - \big [ g + \beta (t) p_{h \rightarrow m}(t) \big ] U_M(t) \end{aligned}$$

32 $$\begin{aligned} p_{h \rightarrow m}(t)&= p_0 \Bigg ( \exp \bigg \{ - \int ^t_0 \beta (\tau ) p_{m \rightarrow h} \frac{I_M(\tau )}{P_H} \bigg [ 1 - \frac{1 - (1-p_{tb}) p_{{p}, I}(t - \tau )}{1 + \nu (1-p_{tb}) p_{{h}, I}(t-\tau )} \bigg ] d \tau \bigg \} \nonumber \\&\quad - \exp \bigg \{ - \int ^t_0 \beta (\tau ) p_{m \rightarrow h} \frac{I_M(\tau )}{P_H} \bigg [ 1 - \frac{1 - (1-p_{tb}) \cdot p_{p, I}(t-\tau ) - p_{p, A}(t-\tau ) }{1 + \nu (1-p_{tb}) p_{h, I}(t-\tau ) + \nu p_{h, A}(t-\tau )} d \tau \bigg \} \Bigg ) \end{aligned}$$

with initial condition $I_M(0), L_M(0), U_M(0) \ge 0$, where we have used Eqs. (16) and (22)–(24). Recall that

- $p_{h, A}(x)$ denotes the probability that a hypnozoite has activated to give rise to a relapse that is ongoing time *x* after inoculation (Eq. (35));
- $p_{h, I}(x)$ denotes the probability that immune memory has been gained (following the clearance of a relapse) time *x* after a hypnozoite is established in the liver (Eq. (36));
- $p_{p, A}(x)$ denotes the probability that a primary infection is ongoing time *x* after onset (Eq. (10));
- $p_{p, I}(x)$ denotes the probability that immune memory has been gained time *x* after the onset of a primary infection (Eq. (10)).

Interpretations for each transmission/within-host parameter are detailed in Table 1.

The system of IDEs given by Eqs. (29)–(32) couples the expected host and vector dynamics, whilst concurrently capturing the accrual of the hypnozoite reservoir (either short- or long-latency strains); superinfection; transmission-blocking immunity and fluctuations in the mosquito population size (due to seasonality or the implementation of vector-based control measures). Given the human population is both immune- and infection-naive at time zero (initial condition (28)), the time evolution of the FORI and the probability of human-to-mosquito transmission per bloodmeal are the same under the system of IDEs given by Eqs. (29)–(32), and the countable system of ODEs given by Eqs. (21)–(24). In the absence of time variation in the mosquito bite $\beta (t)=\beta$ and death $\omega (t)=\omega$ rates, the bifurcation parameter $R_0$ (Eq. (27)) governing the existence of endemic equilibria, and the stationary solutions to the system of ODEs given by Eqs. (21)–(24), therefore also apply to the reduced system of IDEs given by Eqs. (29)–(32).

While the quantities $I_M(t)$, $L_M(t)$, $U_M(t)$ and $p_{h \rightarrow m}(t)$ are sufficient to couple host and vector dynamics, we ultimately seek to characterise population-level distributions for quantities of epidemiological interest. We note, however, that the complete population-level distribution of superinfection, immunity and hypnozoite states can be recovered conditional on the FORI using the results derived in Mehra et al. (2022). To reiterate the premise of the hybrid approximation, the population-level transmission models discussed here have been constructed by regarding the within-host probabilistic distribution as being the same as the population-level frequency distribution (Henry 2020). Given the time evolution of the FORI $\lambda (t)=\beta (t) p_{m \rightarrow h} I_M(t)/P_H$ derived from the system of IDEs given by Eqs. (29)–(32), we can recover population-level distributions for quantities of epidemiological interest using the formulae derived in Mehra et al. (2022). Granted the human population is initially infection- and immune-naive, the time evolution of these population-level distributions will be the same under the countable system of ODEs given by Eqs. (21)–(24), and the reduced system of IDEs given by Eqs. (29)–(32), since the time evolution the FORI itself is the same under this initial condition.

The methodology adopted here uses an integral system, within which we can enforce time-dependence in the bite rate per mosquito $\beta (t)$ and the mosquito birth rate $\omega (t)$. In Sect. 3.3.1 below, we present illustrative results for two scenarios: a constant transmission setting, where all transmission parameters are fixed (Sect. 3.3.1.1); and a seasonal transmission setting, with a sinusoidal mosquito birth rate $\omega (t)$ (Sect. 3.3.1.2). Vector-based control interventions represent a natural extension (Le Menach et al. 2007; Griffin 2010; White 2018a), but are not presented here.

#### Illustrative results for the reduced system of IDEs

To recover transient host and vector dynamics, we solve the system of IDEs given by Eqs. (29) and (32) numerically, using Euler’s method (for the ODEs given by Eqs. (29)–(31)) and the trapezoidal rule (for the integral given by Eq. (32)) with a fixed time step; this procedure is a variation of the algorithm proposed by Anwar et al. (2022). As a function of the FORI derived from Eqs. (29) and (32), we recover the time evolution of several quantities of epidemiological interest. Relevant formulae (as per (Mehra et al. (2022)) are provided in Appendix B, including:

- the mean and variance for the size of the (non)-latent hypnozoite reservoir (Eqs. (37) and (38));
- the PMF for the number of co-circulating blood-stage broods (Eqs. (39)–(41));
- the relapse rate conditional on the blood-stage infection status (Eqs. (44)–(47));
- the distribution of immunity, as quantified by the mean and variance of the discrete immunity levels (Eqs. (48) and (49));
- the prevalence of clinical infection (Eq. (15)).

Results are generated under the parameter values detailed in Table 1.

**Fig. 6:**
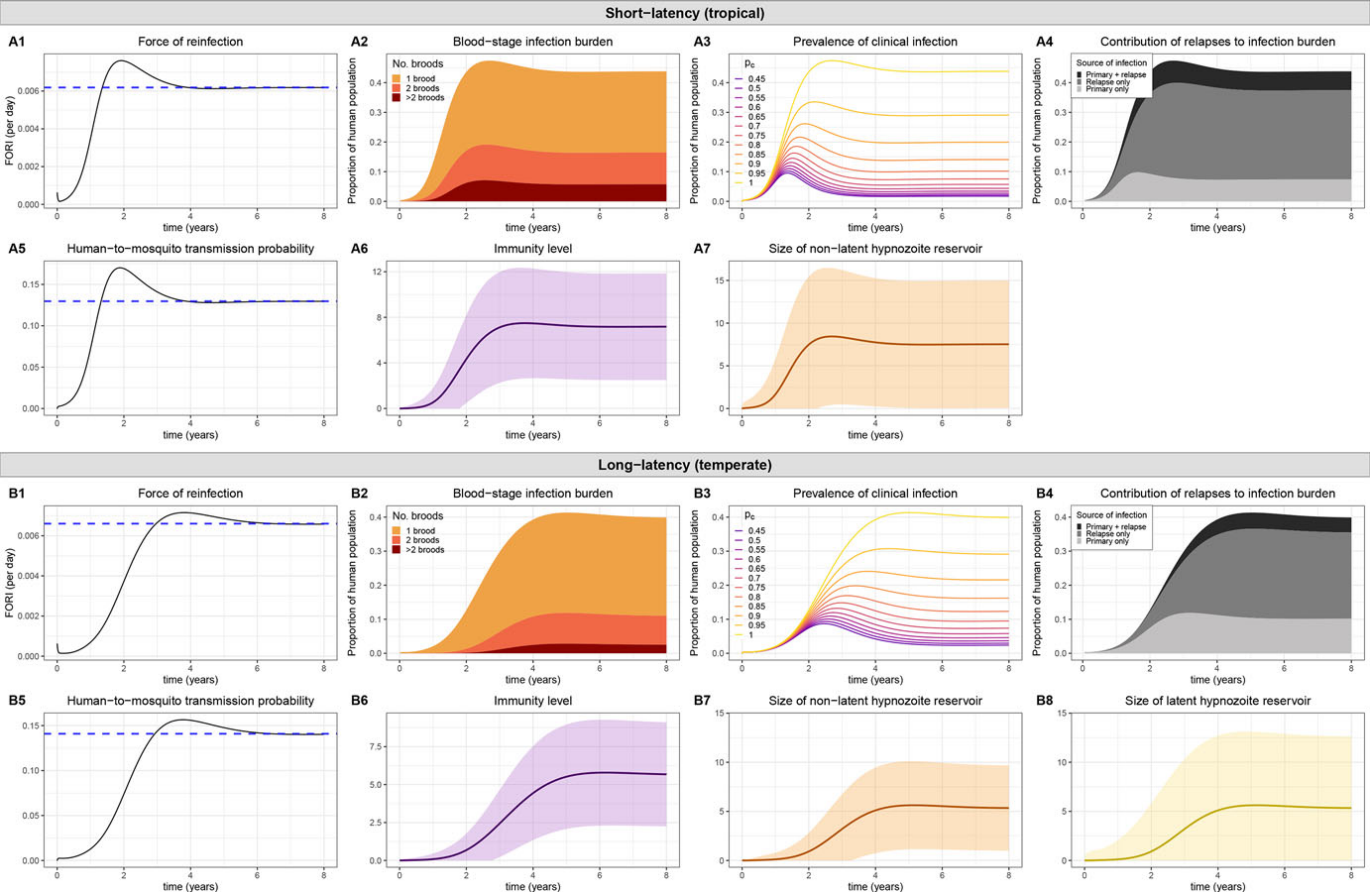
Transient host and vector dynamics for **A** short-latency ($k=0$) and **B** long-latency ($k=2$, $\delta =1/100$ day^-1^) strains. Here, we consider a constant transmission setting with $\beta (t) = \beta$ and $\omega (t) = g$. Model parameters $\alpha , \mu , \gamma , \nu , w, g, \eta , \beta , p_{m \rightarrow h}, p_{tb}$ are given in Table 1, with $p_0 = 0.65$. At time zero, we assume that the human population is both infection- and immune-naive, with $I_M(0)/P_H = 0.012, L_M(0)/P_H = 0, U_M(0)/P_H = 1.2$. We numerically solve the system over a period of 8 years, with a fixed time step of 0.1 days. The (**A1**/**B1**) FORI $\beta p_{m \rightarrow h} \frac{I_M(t)}{P_H}$ and (**A5**/**B5**) probability of human-to-mosquito tranmission $p_{h \rightarrow m}(t)$ are governed by the system of IDEs given by Eqs. (29) and (32). Endemic equilibrium solutions for the FORI $\beta p_{m \rightarrow h} \frac{I_M*}{P_H}$ and human-to-mosquito transmission probability $p^*_{h \rightarrow m}$, given by the non-trivial solution to Eqs. (26) and (25), are indicated with dashed blue lines. All other quantities are calculated as a function of the numerical solution for the FORI $\beta p_{m \rightarrow h} \frac{I_M(t)}{P_H}$, including $\bullet$ (**A2**/**B2**): the PMF for the number co-circulating parasite broods (Eqs. (39)–(41)) $\bullet$ (**A3**/**B3**): the prevalence of clinical infection (Eq. (15)), with $p_c$ ranging from 0.45 to 1 in increments of 0.05 $\bullet$ (**A4**/**B4**): the respective contributions of relapses and primary infections to the burden of blood-stage infection $\bullet$ (**A6**/**B6**): the mean immunity level (Eq. (48)) (shading indicates one standard deviation (Eq. (49)) above and below the mean) $\bullet$ (**A7**/**B7**, **A8**/**B8**): the expected size of the (non)-latent hypnozoite reservoir (Eq. (37)) (shading indicates one standard deviation (Eq. (38)) above and below the mean)

**3.3.1.1 Non-seasonal transmission** We begin by considering host and vector dynamics in the absence of seasonality (Fig. 6). At time zero, we consider the introduction of several infected mosquitoes into an (blood- and liver-stage) infection and immune naive human population. Predicted endemic equilibrium solutions, given by the unique non-trivial solution to Eqs. (26) and (25), are shown with dashed blue lines for the FORI (Figs. 6A1/B1) and the immunity-modulated probability of human-to-mosquito transmission (Figs. 6A5/B5).

Illustrative dynamics for short latency strains ($k=0$) are shown in Fig. 6A. The low initial level of infection in the mosquito population constrains the transmission intensity at early time points. Prior to the acquisition of extensive transmission-blocking immunity—with relatively low immunity levels harboured for a year following the introduction of infected mosquitoes into an immune-naive human population (Fig. 6A6)—human-to-mosquito transmission remains comparatively unconstrained, leading to a sustained increase in the FORI (Fig. 6A1), and consequently, the blood-stage infection burden (Fig. 6A2) and the size of the hypnozoite reservoir (Fig. 6A7). A pronounced rise in the prevalence of blood-stage infection during this early period leads to an increase in both the probability of human-to-mosquito transmission (Fig. 6A5) and the prevalence of clinical infection (Fig. 6A3). Intensified transmission, however, is accompanied by the sustained acquisition of immunity (Fig. 6A6), which eventually mitigates the probability of human-to-mosquito transmission (Fig. 6A5), leading to a reduction in the FORI (Fig. 6A5), as well as a slight reduction in the burden of (clinical) blood-stage infection (Fig. 6A2, A3). These transient effects eventually subside, and for the set of parameters considered here, the predicted endemic equilibrium (obtained by numerically solving Eqs. (25)–(26), and indicated with dotted lines blue lines) is reached within approximately 4 years.

Analogous results for long-latency strains ($k>0$) are shown in Fig. 6B. As a consequence of the enforced hypnozoite dormancy period (with expected duration $k/\delta = 200$ days and standard deviation $\sqrt{k}/\delta = 100 \sqrt{2}$ days), the non-latent hypnozoite reservoir remains limited in size for approximately one year (Fig. 6B7); as such, single-brood primary infections dominate the infection burden for an extended period of time (Fig. 6B2, B4) relative to short-latency strains. In tandem with the emergence of hypnozoites from dormancy, relapses eventually drive up the burden of (clinical) blood-stage infection (Fig. 6B2, B4). As for short-latency strains ($k=0$), the acquisition of transmission-blocking immunity eventually mitigates onward human-to-mosquito transmission (Fig. 6B5), leading to a slight reduction in transmission intensity before the predicted endemic equilibrium (obtained by numerically solving Eqs. (25) and (26), and indicated with dotted lines blue lines) is reached six years after the introduction of infected mosquitoes into an infection- and immune-naive human population.

**Fig. 7:**
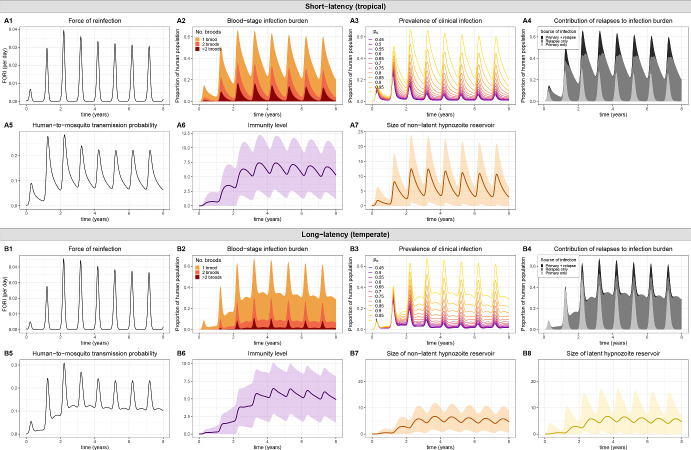
Transient host and vector dynamics for **A** short-latency ($k=0$) and **B** long-latency ($k=2$, $\delta =1/100$ day^-1^) strains. Here, we consider a seasonal transmission setting with $\beta (t) = \beta$ and $\omega (t) = g \big [ \sin (\frac{2 \pi t}{365} + \frac{3 \pi }{4}) + 1 \big ]$. Model parameters $\alpha , \mu , \gamma , \nu , w, g, \eta , \beta , p_{m \rightarrow h}, p_{tb}$ are given in Table 1, with $p_0 = 0.65$ At time zero, we assume that the human population is both infection- and immune-naive, with $I_M(0)/P_H = 0.012, L_M(0)/P_H = 0, U_M(0)/P_H = 1.2$. We numerically solve the system over a period of 8 years, with a fixed time step of 0.02 days. The (**A1**/**B1**) FORI $\beta p_{m \rightarrow h} \frac{I_M(t)}{P_H}$ and (**A5**/**B5**) probability of human-to-mosquito tranmission $p_{h \rightarrow m}(t)$ are governed by the system of IDEs given by Eqs. (29) and (32). All other quantities are calculated as a function of the numerical solution for the FORI $\beta p_{m \rightarrow h} \frac{I_M(t)}{P_H}$, including $\bullet$ (**A2**/**B2**): the PMF for the number co-circulating parasite broods (Eqs. (39)–(41)). $\bullet$ (**A3**/**B3**): the prevalence of clinical infection (Eq. (15)), with $p_c$ ranging from 0.45 to 1 in increments of 0.05. $\bullet$ (**A4**/**B4**): the respective contributions of relapses and primary infections to the burden of blood-stage infection. $\bullet$ (**A6**/**B6**): the mean immunity level (Eq. (48)) (sharing indicates one standard deviation (Eq. (49)) above and below the mean). $\bullet$ (**A7**/**B7**, **A8**/**B8**): the expected size of the (non)-latent hypnozoite reservoir (Eq. (37)) (shading indicates one standard deviation (Eq. (38)) above and below the mean)

**3.3.1.2 Seasonal transmission** To allow for seasonality, arising, for instance, from external climactic variation, we impose sinusoidal forcing (with period one year) on the mosquito birth rate. Illustrative dynamics for short-latency ($k=0$) and long-latency ($k>0$) strains are shown in Fig. 7A, B respectively. With the imposition of seasonal forcing, infection levels within both humans and mosquitoes exhibit oscillations (with period one year) that eventually stabilise around a steady mean. The nature of these oscillations within a season, however, varies between short- and long-latency strains. Oscillations in the FORI (Fig. 7A1/B1) are driven strongly by seasonal fluctuations in the size of the mosquito population. For short-latency strains ($k=0$), the burden of (clinical) blood-stage infection decays monotonically between seasonal peaks (Fig. 7A2, A3) as the hypnozoite reservoir is depleted in light of limited mosquito-to-human transmission (as quantified by the FORI, Fig. 7A1). For long-latency strains, yearly maxima in the burden of (clinical) blood-stage infection (Fig. 7B2, B3) likewise coincide with seasonal peaks in the FORI (Fig. 7B1), with primary infections contributing to the majority of the blood-stage infection burden during these periods of intensified mosquito-to-human transmission (Fig. 7B4). As a consequence of the hypnozoite dormancy period—which introduces a delay between periods of intensified mosquito-to-human transmission and elevated relapse risk (as quantified through the size of the non-latent hypnozoite reservoir, Fig. 7B7)—we observe biphasic infection dynamics, whereby the burden of (clinical) blood-stage infection exhibits a second, smaller peak approximately 6 months after the yearly maximum (Fig. 7B2, B3) driven by relapses (Fig. 7B4) as hypnozoites established during the seasonal peak of mosquito-to-human transmission emerge from dormancy. As such, we predict that hypnozoite dormancy sustains the burden of blood-stage infection between seasonal peaks in the FORI, in line with the hypothesis that long-latency phenotypes evolved in temperate regions to sustain transmission despite of limited mosquito breeding during the winter season (White 2016).

## Discussion

The interplay between the hypnozoite reservoir, superinfection and acquired immunity is a key aspect of the epidemiology of *P. vivax*. Here, we have proposed novel hybrid transmission models for *P. vivax*, accounting for hypnozoite accrual, (blood-stage) superinfection and the acquisition of transmission-blocking and antidisease immunity. To capture within-host dynamics as a function of the FORI, we have extended the open network of infinite server queues constructed in Mehra et al. (2022) to embed a discretised version of the antibody model we introduced in Mehra et al. (2021). By deriving the joint PGF for the state of the queueing network (Eq. (11)), we have obtained an analytic description of within-host dynamics in a general transmission setting. To couple host and vector dynamics, we have adopted the hybrid approximation of Nåsell (2013) under which probabilistic within-host distributions are cast as expected population-level proportions (Henry 2020). We have thus recovered a deterministic compartmental model, comprising a countably infinite system of ODEs (Eqs. (21) and (24)), which can be viewed as a natural extension of the Ross-Macdonald framework. For a simpler system with countably many states, we demonstrated the equivalence of the hybrid approximation to the functional law of large numbers (Barbour and Luczak 2012) for an appropriate Markov population process in Mehra et al. (2023).

We have drawn on distributions derived at the within-host level (Mehra et al. 2022) to characterise both the transient and steady state behaviour of this compartmental model. In particular, following the approach we developed in Mehra et al. (2023), we have derived a reduced system of IDEs governing the time evolution of the number of (un)infected and latent mosquitoes; and the immunity-modulated probability of human-to-mosquito transmission (Eqs. (29)–(32)). As a function of the FORI predicted under this reduced system of IDEs—which is equivalent to the complete compartmental model granted the human population is initially immune- and infection-naive—we have recovered complete population-level distributions for various quantities of epidemiological interest, using the formulae derived in Mehra et al. (2022) (see Appendix B). By drawing on the within-host queueing models we introduced in Mehra et al. (2021, 2022), and the construction developed in Mehra et al. (2023), we have circumvented the practical constraints that have previously limited the tractability of hypnozoite density models (White 2018a).

Our model, to the best of our knowledge, provides the most complete description of superinfection, immunity and hypnozoite dynamics for *P. vivax* thus far, while remaining readily amenable to numerical solution and analysis. In Mehra et al. (2023), we developed a framework to capture the dynamics of (short-latency) hypnozoite accrual and superinfection, addressing a gap in the literature with respect to the rigorous analysis of superinfection; we have extended this framework in the present manuscript to allow for acquired immunity and long-latency phenotypes. The joint population-level dynamics of the hypnozoite reservoir and acquired immunity have been previously examined by White (2018a). The construction of White (2018a) is predicated on a continuous age- and exposure-dependent immunity level, which is subsequently mapped (using Hill functions) to correlates of antidisease immunity (that is, a reduced probability of clinical infection) and antiparasite immunity (including accelerated parasite clearance and mitigated parasite densities, manifesting in a reduced probability of detection via light microscopy). Here, we have instead considered a discretised exposure-dependent immunity level, which we have mapped to correlates of antidisease immunity and transmission-blocking immunity (that is, a reduced probability of human-to-mosquito transmission) under the assumption of geometric decay. While White (2018a) explicitly account for treatment, and age structure and heterogeneity in the human population, we have restricted our attention to a homogeneous human population in the absence of treatment and age structure. Unlike White (2018a), however, we monitor hypnozoite densities rather than broods (thereby capturing variation in parasite inocula across bites), in addition to the population-level distribution of superinfection; our framework can also be employed to analyse long-latency hypnozoite strains, unlike the framework of White (2018a) which is restricted to short-latency strains.

While most previous hypnozoite ‘batch’ and ‘density’ models (White et al. 2014; White 2018a; Anwar et al. 2022) have relied on numerical solution to characterise steady state properties, our sensitivity analyses are informed by the within-host distributions derived in Mehra et al. (2022). We recover a threshold phenomenon for the hybrid model, deriving a bifurcation parameter (Eq. (27)) governing the existence of endemic equilibria. In the absence of transmission-blocking immunity ($p_{tb} = 1$) and mosquito latency ($1/\eta = 0$), we were able to perform an asymptotic stability analysis in Mehra et al. (2023) for the first-order IDE governing the time-evolution of the FORI using the stability criterion of Brauer (1978); the imposition of transmission blocking immunity ($p_{tb} < 1$) or mosquito latency ($1/\eta > 0$) yields a higher-order system of IDEs governing the FORI, for which we are unaware of asymptotic stability criteria.

The transient and stationary results presented in this manuscript are underpinned by analyticity at the within-host level, which, in turn, is predicated on the assumption that each hypnozoite/infection is governed by an independent stochastic process. The assumption of independent, spontaneous hypnozoite activation, as implemented by White et al. (2014), is critical to our construction: external triggers of hypnozoite activation (e.g. febrile illness, arising from parasitic or bacterial infections (Dennis Shanks and White 2013), particularly *P. falciparum* (Commons 2019)) necessarily introduce synchronicity between activating hypnozoites, thereby violating the assumption of independent hypnozoite behaviour. Our model does not readily accommodate interactions between concurrent hypnozoites/infections, for instance, competition between co-circulating parasite broods (De Roode 2005). Antiparasite immunity (manifest in the modulation parasite clearance rates (White 2017)) and pre-erythrocytic immunity (Mueller et al. 2013), which render hypnozoite/infection dynamics dependent on the infection history, are likewise intractable. On a population-level, our model is constrained by the assumption of homogeneity for the human population. We do not account for age structure or demography within the human population; heterogeneity in the risk of relapse and immunity levels across individuals is driven purely by stochastic fluctuations, rather than differences in the time over which the hypnozoite reservoir has been accrued and immunity has been acquired.

Our formulation of immunity, moreover, is non-mechanistic and subject to a number of simplifying assumptions. Adopting a discretised version of the model presented in Mehra et al. (2021), we assume that the clearance of each blood-stage infection is accompanied by an immunity boost with unit magnitude and an exponentially-distributed lifetime. Empirical characterisation of antibody titres, however, has revealed substantial heterogeneity in the magnitude of antibody boosts across successive infections, and the temporal distribution of antibody boosts associated with different antigens (White et al. 2014). A key omission in our model is strain specificity; while homologous challenge yields a strong immune response, immune protection following heterologous challenge is contingent on the degree of cross-reactivity between strains (Mueller et al. 2013). As such, the discretised immunity level considered here largely serves as a proxy for ‘recent’ exposure to blood-stage infection, with the immune decay parameter *w* governing the time scale on which immunity is retained.

Nonetheless, in capturing the interplay between hypnozoite accrual, superinfection and acquired immunity—and providing, to the best of our knowledge, the most complete population-level distributions for a range of epidemiological values—our model provides insights into important, but poorly understood, epidemiological features of *P. vivax*, with natural extensions to explore the consequences of control and elimination strategies.

## Data Availability

Data sharing not applicable to this article as no datasets were generated or analysed during the current study.

## State probabilities for a single hypnozoite

Here, we provide solutions to the system of ODEs given by Eqs. (2)–(6). We have previously solved related models in Mehra et al. (2020, 2022):

- In Mehra et al. (2020), we analysed the activation-clearance model proposed by White et al. (2014), but did not model the dynamics of blood-stage infection following each hypnozoite activation event (that is, states *A*, *I* and *C* were not distinguished).
- In Mehra et al. (2022), we constructed a relapse-clearance model, extending the model proposed in White et al. (2014) (and solved in Mehra et al. (2020)) to accommodate a blood-stage infection (of exponentially-distributed duration) following hypnozoite activation, but did not capture the acquisition/waning of immunity (that is, states *I* and *C* were not distinguished).

As in Mehra et al. (2020) and Mehra et al. (2022), we solve the system of ODEs successively using integration by parts, drawing on standard integral number 2.321.2 of Jeffrey and Zwillinger (2007), to give:

33 $$\begin{aligned} p_{h,m}(t) =&\frac{(\delta t)^{m-1}}{(m-1)!}e^{-(\mu +\delta )t} \text { for } m \in [1, k] \end{aligned}$$

34 $$\begin{aligned} p_{h,NL}(t) =&\frac{\delta ^{k}}{(\delta - \alpha )^{k}} \Bigg [ e^{-(\mu + \alpha )t} - e^{-(\mu + \delta )t} \sum ^{k-1}_{j=0} \frac{t^j}{ j!} (\delta - \alpha )^{j} \Bigg ] \end{aligned}$$

35 $$\begin{aligned} p_{h,A}(t) =&\frac{\alpha \delta ^{k}}{(\delta - \alpha )^{k}} \Bigg [\frac{e^{-(\mu + \delta )t}}{\mu + \delta - \gamma } \Bigg \{ \sum ^{k-1}_{i=0} t^i \bigg [ \frac{(\mu -\gamma + \delta )^{i}}{i!} \sum ^{k-1}_{j=i} \Big ( \frac{\delta - \alpha }{\mu - \gamma + \delta } \Big )^j \bigg ] \Bigg \} - \frac{e^{-(\mu + \alpha )t}}{\mu - \gamma + \alpha } \Bigg ] \nonumber \\&+ \frac{\alpha }{\alpha + \mu - \gamma } \Big ( \frac{\delta }{\delta + \mu - \gamma } \Big )^{k} e^{-\gamma t} \end{aligned}$$

36 $$\begin{aligned} p_{h,I}(t) =&\frac{\alpha }{\alpha + \mu - \gamma } \Big ( \frac{\delta }{\delta - \alpha } \Big )^k \frac{\gamma }{\alpha + \mu -w } e^{-(\mu + \alpha )t} \nonumber \\&+ \frac{\gamma }{w - \gamma } \frac{\alpha }{\alpha + \mu - \gamma } \Big ( \frac{\delta }{\delta + \mu - \gamma } \Big )^{k} e^{-\gamma t} \nonumber \\&- \frac{\gamma \alpha \delta ^k}{(\delta - \alpha )^k} \frac{e^{-(\mu + \delta ) t}}{(\mu + \delta - \gamma )(\mu + \delta - w)} \nonumber \\&\quad \Bigg \{ \sum ^{k-1}_{\ell =0} t^\ell \Bigg [ \frac{(\mu + \delta - w)^{\ell }}{\ell !} \sum ^{k-1}_{i=\ell } \Big ( \frac{\mu +\delta -\gamma }{\mu +\delta -w} \Big )^i \sum ^{k-1}_{j=i} \Big ( \frac{\delta -\alpha }{\mu + \delta - \gamma }\Big )^j \Bigg ] \Bigg \}\nonumber \\&+ \frac{\gamma }{\gamma -w} \frac{\alpha }{\alpha + \mu - w} \Big ( \frac{\delta }{\delta + \mu - w} \Big )^k \end{aligned}$$

Solutions for $p_{h,m}(t)$, $m\in [1, k]$ and $p_{h,NL}(t)$ were intially presented in Mehra et al. (2020), while solutions for $p_{h,A}(t)$ were initially derived in Mehra et al. (2022).

## Population-level distributions for quantities of epidemiological interest

As a function of the FORI $\lambda (t)$ in the interval $\tau \in [0, t)$, we can recover distributions for various quantities of epidemiological interest at time *t* using the results derived in Mehra et al. (2022). In the case of the open network of infinite server queues described in Sect. 2, we assume a functional form for $\lambda (t)$, and interpret these quantities as probabilistic distributions at the within-host level. In the context of the hybrid transmission models constructed in Sect. 3, where we capture the time evolution of $\lambda (t)$ by coupling host and vector dynamics, these quantities are instead interpreted as population-level proportions (Henry 2020). We adopt the same notation for both situations, with $N_s(t)$ denoting the marginal distribution for the number of hypnozoites/infections in state $s \in S$ at time *t* on either the within-host or population-level.

### Size of the (non)-latent hypnozoite reservoir

The expected size of the (non)-latent hypnozoite reservoir at time *t*

37 $$\begin{aligned} {{\,\mathrm{\mathbbm {E}}\,}}[ N_{(N)L}(t) ]&= \nu \int ^t_0 \lambda (\tau ) p_{h,(N)L}(t-\tau ) d\tau \end{aligned}$$

and associated variance

38 $$\begin{aligned} \text {Var} [ N_{(N)L}(t)]&= \nu \int ^\infty _0 \lambda (\tau ) p_{h,(N)L}(t-\tau ) \big [ 1 + 2 \nu p_{h,(N)L}(t-\tau ) \big ] d \tau \end{aligned}$$

follow from Equations (39) and (74) of Mehra et al. (2022). Complete distributions for $S_{(N)L}(t)$ can be recovered using Equations (75) and (76) of Mehra et al. (2022). For short-latency strains ($k=0$), a constant FORI $\lambda (\tau ) = \lambda ^*$ yields the steady state distribution

$$\begin{aligned} N^*_{NL} \sim \text {NegativeBinomial} \Big ( \frac{\nu }{1+\nu }, \frac{\lambda ^*}{\alpha + \mu } \Big ), \end{aligned}$$

as shown in Equation (36) of Mehra et al. (2022).

### Blood-stage infection status

Equations (82) and (83) of Mehra et al. (2022) yield the complete population-level distribution for the number of co-circulating parasite broods. Here, we state the respective probabilities of carrying $n=0,1,2$ broods of co-circulating parasites at time *t*:

39 $$\begin{aligned}&P(N_A(t) + N_P(t) = 0)\nonumber \\&\quad = \exp \bigg \{ -\int ^t_0 \lambda (\tau ) \frac{p_{p,A}(t-\tau ) + \nu p_{h,A}(t-\tau )}{1 + \nu p_{h,A}(t-\tau )} d \tau \bigg \} \end{aligned}$$

40 $$\begin{aligned}&P(N_A(t) + N_P(t) = 1)\nonumber \\&\quad = \bigg ( \int ^t_0 \lambda (\tau ) \frac{p_{p,A}(t-\tau ) + \nu p_{h,A}(t-\tau )}{1 + \nu p_{h,A}(t-\tau )} d \tau \bigg ) \nonumber \\&\qquad \times \exp \bigg \{ -\int ^t_0 \lambda (\tau ) \frac{p_{p,A}(t-\tau ) + \nu p_{h,A}(t-\tau )}{1 + \nu p_{h,A}(t-\tau )} d \tau \bigg \} \end{aligned}$$

41 $$\begin{aligned}&P(N_A(t) + N_P(t) = 2)\nonumber \\&\quad = \frac{1}{2} \bigg [ \bigg ( \int ^t_0 \lambda (\tau ) \frac{p_{p,A}(t-\tau ) + \nu p_{h,A}(t-\tau )}{1 + \nu p_{h,A}(t-\tau )} d\tau \bigg )^2 \nonumber \\&\qquad + 2 \int ^t_0 \lambda (\tau ) \frac{\nu p_{h,A}(t-\tau ) \big ( p_{p,A}(t-\tau ) + \nu p_{h,A}(t-\tau ) \big ) }{\big (1 + \nu p_{h,A}(t-\tau ) \big )^3} d\tau \bigg ] \nonumber \\&\qquad \times \exp \bigg \{ -\int ^t_0 \lambda (\tau ) \frac{p_{p,A}(t-\tau ) + \nu p_{h,A}(t-\tau )}{1 + \nu p_{h,A}(t-\tau )} d \tau \bigg \}. \end{aligned}$$

### Relapse rate conditional on blood-stage infection status

The joint PGF for the size of the non-latent hypnozoite reservoir $N_{NL}(t)$ and the number of co-circulating parasite broods $M_I(t):= N_A(t) + N_P(t)$ can be written

42 $$\begin{aligned}&{{\,\mathrm{\mathbbm {E}}\,}}\big [ x^{NL(t)} y^{M_I(t)} ]\nonumber \\&\quad = G(z_1 = 1, \dots , z_k=1, z_{NL}=x, z_A=y, z_C=1, z_D=1, z_P=y)\nonumber \\&\quad = \exp \Bigg \{ -\int ^t_0 \lambda (\tau ) \Bigg [ 1 - \frac{1 + (y-1) p_{p,A}(t-\tau ) }{1 + \nu ( 1 - x) p_{h,NL}(t) + \nu (1-y) p_{h,A}(t)} \Bigg ] d \tau \Bigg \} \end{aligned}$$

where we have used Eq. (11).

The (unconditional) relapse rate, which is proportional to the expected size of the non-latent hypnozoite reservoir, can be written

43 $$\begin{aligned} r(t)&:= \alpha {{\,\mathrm{\mathbbm {E}}\,}}[N_{NL}(t)] = \alpha \nu \int ^t_0 \lambda (\tau ) p_{h,NL}(t-\tau ) d \tau \end{aligned}$$

as in Equation (39) of Mehra et al. (2022).

Conditional on the absence of blood-stage infection ($M_I(t)=0$), the relapse rate is given by

44 $$\begin{aligned} r_0(t)&:= \alpha {{\,\mathrm{\mathbbm {E}}\,}}[N_{NL}(t) | M_I(t) = 0] = \frac{\frac{\partial }{\partial x} {{\,\mathrm{\mathbbm {E}}\,}}\big [ x^{NL(t)} y^{M_I(t)} ]|_{x=1, y=0}}{{{\,\mathrm{\mathbbm {E}}\,}}\big [ x^{NL(t)} y^{M_I(t)} ]|_{x=1, y=0}}\nonumber \\&= \alpha \int ^t_{0} \lambda (\tau ) \frac{ \nu p_{h,NL}(t-\tau ) \big ( 1 - p_{p, A}(t-\tau ) \big )}{\big ( 1 + \nu p_{h,A}(t-\tau ) \big )^2} d \tau \end{aligned}$$

as in Equation (78) of Mehra et al. (2022).

Likewise, by Xekalaki (1987), we recover the relapse rate conditional on a single-brood blood-stage infection ($M_I(t)=1$),

45 $$\begin{aligned} r_1(t)&:= \alpha {{\,\mathrm{\mathbbm {E}}\,}}[N_{NL}(t) | M_I(t) = 1] = \frac{\frac{\partial ^2}{\partial x \partial y} {{\,\mathrm{\mathbbm {E}}\,}}\big [ x^{NL(t)} y^{M_I(t)} ]|_{x=1, y=0}}{\frac{\partial }{\partial y}{{\,\mathrm{\mathbbm {E}}\,}}\big [ x^{NL(t)} y^{M_I(t)} ]|_{x=1, y=0}} \nonumber \\&= \alpha \Bigg [ \frac{\int ^t_0 \lambda (\tau ) \frac{\nu p_{h,NL}(t-\tau ) [ 2 \nu p_{h,A}(t-\tau ) + p_{p,A}(t-\tau ) ( 1 - \nu p_{h,A}(t-\tau )) ]}{ (1 + \nu p_{h,A}(t-\tau ) )^3} d \tau }{\int ^t_0 \lambda (\tau ) \frac{\nu p_{h,A}(t-\tau ) + p_{p,A}(t-\tau )}{ (1 + \nu p_{h,A}(t-\tau ) )^2} d \tau } \nonumber \\&\qquad \qquad + \int ^t_{0} \lambda (\tau ) \frac{ \nu p_{h,NL}(t-\tau ) \big ( 1 - p_{p, A}(t-\tau ) \big )}{\big ( 1 + \nu p_{h,A}(t-\tau ) \big )^2} d \tau \Bigg ] \end{aligned}$$

as well as a double-brood blood-stage infection ($M_I(t) = 2$)

46 $$\begin{aligned} r_2(t)&:= \alpha {{\,\mathrm{\mathbbm {E}}\,}}[N_{NL}(t) | M_I(t) = 2] = \frac{\frac{\partial ^3}{\partial x \partial y^2} {{\,\mathrm{\mathbbm {E}}\,}}\big [ x^{NL(t)} y^{M_I(t)} ]|_{x=1, y=0}}{\frac{\partial ^2}{\partial y^2}{{\,\mathrm{\mathbbm {E}}\,}}\big [ x^{NL(t)} y^{M_I(t)} ]|_{x=1, y=0}} \nonumber \\&= \alpha \Bigg [ \Bigg (\int ^t_0 \lambda (\tau ) \frac{2 \nu ^2 p_{h,NL}(t-\tau ) p_{h,A}(t-\tau ) [3 \nu p_{h,A}(t-\tau ) + p_{p,A}(t-\tau ) ( 2 - \nu p_{h,A}(t-\tau )) ] }{(1 + \nu p_{h,A}(t-\tau ))^4} d \tau \nonumber \\&\quad + 2 \Big [ \int ^t_0 \lambda (\tau ) \frac{\nu p_{h,A}(t-\tau ) + p_{p,A}(t-\tau )}{ (1 + \nu p_{h,A}(t-\tau ) )^2} d \tau \Big ] \Big [ \int ^t_0 \lambda (\tau ) \frac{\nu p_{h,NL}(t-\tau ) [ 2 \nu p_{h,A}(t-\tau ) {+} p_{p,A}(t-\tau ) ( 1 {-} \nu p_{h,A}(t{-}\tau )) ]}{ (1 + \nu p_{h,A}(t-\tau ) )^3} d \tau \Big ] \Bigg ) \nonumber \\&\quad \times \Bigg ( \int ^t_0 \lambda (\tau ) \frac{2 \nu p_{h,A}(t-\tau ) [\nu p_{h,A}(t-\tau ) + p_{p,A}(t-\tau )}{(1 + \nu p_{h,A}(t-\tau ))^3} d \tau + \Big [ \int ^t_0 \lambda (\tau ) \frac{\nu p_{h,A}(t-\tau ) + p_{p,A}(t-\tau )}{ (1 + \nu p_{h,A}(t-\tau ) )^2} d \tau \Big ]^2\Bigg )^{-1} \nonumber \\&\quad + \int ^t_{0} \lambda (\tau ) \frac{ \nu p_{h,NL}(t-\tau ) \big ( 1 - p_{p, A}(t-\tau ) \big )}{\big ( 1 + \nu p_{h,A}(t-\tau ) \big )^2} d \tau \Bigg ]. \end{aligned}$$

By the law of total expectation, we can recover the relapse rate conditional on a blood-stage infection comprising three or more parasite broods ($M_I(t)>2$):

47 $$\begin{aligned} r_{>2}(t)&:= \alpha {{\,\mathrm{\mathbbm {E}}\,}}[N_{NL}(t) | M_I(t) > 2] \nonumber \\&= \frac{r(t) - r_0(t) \cdot P(M_I(t) = 0) - r_1(t) \cdot P(M_I(t) = 1) - r_2(t) \cdot P(M_I(t) = 2) }{ 1 - P(M_I(t) = 0) - P(M_I(t) = 1) - P(M_I(t) = 2)} \end{aligned}$$

using Eqs. (39)–(41) and (43)–(46).

### Relative contribution of relapses to the infection burden

At time *t*, each individual in the human population has no ongoing primary infections with probability

$$\begin{aligned} P(N_P(t) = 0) = \exp \bigg \{ -\int ^t_0 \lambda (\tau ) p_{p,I}(t-\tau ) d\tau \bigg \} \end{aligned}$$

and no ongoing relapses with probability

$$\begin{aligned} P(N_A(t) = 0) = \exp \bigg \{ -\int ^t_0 \lambda (\tau ) \frac{\nu p_{h,A}(t-\tau )}{1 + \nu p_{h,A}(t-\tau )} d\tau \bigg \} \end{aligned}$$

as in Equations (40) and (41) of Mehra et al. (2022). The relative contribution of relapses to the infection burden (that is, the proportion of blood-stage infections that encompass at least one relapse) follows readily from the quantities $P(N_P(t) = 0)$, $P(N_A(t) = 0)$ and $P(N_A(t) = N_P(t) = 0)$ (that is, the probability that each individual has neither a primary infection, nor a relapse, at time *t*).

### Distribution of immunity

From the joint PGF given by Eq. (11), we recover the PGF governing the population-level distribution of immunity *I*(*t*) at time *t*

$$\begin{aligned} {{\,\mathrm{\mathbbm {E}}\,}}\big [ z^{N_I(t)} ]&= G(t, z_1=1, \dots , z_k=1, z_{NL}=1, z_A{=}1, z_D{=}1, z_C{=}1, z_I{=}z, z_P{=}1)\\&= \exp \bigg \{ - \int ^t_0 a I_M(\tau ) \bigg [ 1 - \frac{1 - (1-z) p_{p,I}(t-\tau )}{1 + \nu p_{h,I}(t-\tau ) (1-z)} \bigg ] d \tau \bigg \}. \end{aligned}$$

We can thus compute the expected immunity level

48 $$\begin{aligned} {{\,\mathrm{\mathbbm {E}}\,}}[ N_I(t) ] =&\frac{\partial {{\,\mathrm{\mathbbm {E}}\,}}\big [ z^{P_I(t)} ] }{\partial z} \Bigg |_{z=1} = \int ^t_0 \lambda (\tau ) \big [ \nu p_{h,I}(t-\tau ) + p_{p,I}(t-\tau ) \big ] dt \end{aligned}$$

in addition to the variance

49 $$\begin{aligned} \text {Var}[ N_I(t) ] =&\frac{\partial ^2 {{\,\mathrm{\mathbbm {E}}\,}}\big [ z^{N_I(t)} ] }{\partial z^2} \Bigg |_{z=1} + \frac{\partial {{\,\mathrm{\mathbbm {E}}\,}}\big [ z^{N_I(t)} ] }{\partial z} \Bigg |_{z=1} - \bigg ( \frac{\partial {{\,\mathrm{\mathbbm {E}}\,}}\big [ z^{N_I(t)} ] }{\partial z} \Bigg |_{z=1} \bigg )^2 \nonumber \\ =&\int ^t_0 \lambda (\tau ) \big [ \nu p_{h,I}(t-\tau ) + p_{p,I}(t-\tau ) \big ] \big [ 1 + 2 \nu p_{h,I}(t-\tau ) \big ] dt. \end{aligned}$$

The complete population-level distribution of immunity can be recovered using Faa di Bruno’s formula and Leibiniz’s integral rule, using a similar approach to Mehra et al. (2022) (see Equations (82) and (83) of Mehra et al. (2022), which have an analogous functional form to that consided here).

## Expected vector dynamics

Here, we characterise the dynamics of the vector population, as a function of the probability of successful human-to-mosquito transmission $p_{h \rightarrow m}(t)$. The structure of the Markovian birth-death process governing the state of the vector population is described in Sect. 3.1.

Denote by $p_{i, l, u}(t)$ the probability that the mosquito population comprises *i* infected, *l* latent and *u* uninfected mosquitoes at time *t*. By the Kolmogorov forward differential equations, it follows that

50 $$\begin{aligned}&\frac{d p_{i,l, u}}{dt} \nonumber \\&\quad = \underbrace{g \big [ -(i + u + l) p_{i,l,u}(t) + (i+1) p_{i+1, l, u}(t) + (u+1) p_{i, l, u+1}(t) + (l+1) p_{i, l+1, u}(t)]}_\text {mosquito death} \nonumber \\&\qquad + \underbrace{\omega (t) \big [ - (i + l + u) p_{i, l, u}(t) + (i + l + u - 1) p_{i, l, u-1}(t) \big ]}_\text {mosquito birth} \nonumber \\&\qquad + \underbrace{\beta (t) p_{h \rightarrow m}(t) \big [ - u p_{i, l, u}(t) + (u + 1) p_{i, l-1, u+1}(t) \big ]}_\text {human-to-mosquito transmission} \nonumber \\&\qquad + \underbrace{\eta \big [ - l p_{i, l, u}(t) + (l + 1) p_{i-1, l+1,u}(t) \big ]}_\text {sporogony complete}. \end{aligned}$$

Define the generating function

$$\begin{aligned} F(z_i, z_l, z_u, t) = \sum ^\infty _{j_i = 0} \sum ^\infty _{j_l = 0} \sum ^\infty _{j_u = 0} p_{j_i, j_l, j_u}(t) z_i^{j_i} z_l^{j_l} z_u^{j_j}. \end{aligned}$$

Using the ODEs given by Eq. (50), we recover a PDE for the generating function *F*

51 $$\begin{aligned} \frac{\partial F}{\partial t} =&+ \big [ g(1-z_i) + \omega (t) z_i (z_u - 1) \big ] \cdot \frac{\partial F}{\partial z_i} \nonumber \\&+ \big [ g(1-z_l) + \omega (t) z_l (z_u - 1) + \eta (z_i - z_l) \big ] \cdot \frac{\partial F}{\partial z_u} \nonumber \\&+ \big [ g(1-z_u) + \omega (t) z_u (z_u - 1) + \beta (t) p_{h \rightarrow m}(t) (z_l - z_u) \big ] \cdot \frac{\partial F}{\partial z_u}. \end{aligned}$$

Denote by $I_M(t)$, $L_M(t)$, $U_M(t)$ the expected number of infected, latent and uninfected mosquitoes respectively at time *t*, that is,

$$\begin{aligned} I_M(t) = \frac{\partial F}{\partial z_i} \Big |_{z_i = z_l = z_u = 1} \qquad L_M(t) = \frac{\partial F}{\partial z_l} \Big |_{z_i = z_l = z_u = 1} \qquad U_M(t) = \frac{\partial F}{\partial z_u} \Big |_{z_i = z_l z_u = 1}. \end{aligned}$$

Then for $s \in \{i, l, u\}$, by differentiating the PDE given by Eq. (51) with respect to $z_s$ and evaluating the resultant expression at $z_i = z_l = z_u = 1$, we recover precisely the system of ODEs given by Eqs. (17)–(19) governing the time evolution of $I_M(t)$, $L_M(t)$, $U_M(t)$ as a function of the probability of human-to-mosquito transmission $p_{h \rightarrow m}(t)$ per bloodmeal. More detailed vector dynamics can be captured in an analogous manner.

## The existence of endemic equilibrium solutions

For notational convenience, let

$$\begin{aligned} A&:= \frac{\beta p_{m \rightarrow h}}{P_H} \int ^\infty _0 \frac{1 - (1-p_{tb}) p_{p, I}(\tau )}{1 + \nu (1-p_{tb}) p_{h, I}(\tau )} - \frac{1 - (1-p_{tb}) p_{p, I}(\tau ) - p_{p, A}(\tau )}{1 + \nu (1-p_{tb}) p_{h, I}(\tau ) + \nu p_{h, A}(\tau )} d \tau \\ C&:= \frac{\beta p_{m \rightarrow h}}{P_H} \int ^\infty _0 1 - \frac{1 - (1-p_{tb}) p_{p, I}(\tau )}{1 + \nu (1-p_{tb}) p_{h, I}(\tau )} d \tau . \end{aligned}$$

From Eqs. (13) and (14), we observe that

$$\begin{aligned} e^{-C I_M^*}&= {{\,\mathrm{\mathbbm {E}}\,}}\big [ p_{tb}^{N_I(t)} \big ] = \sum ^\infty _{i_1=0} \dots \sum ^\infty _{i_k=0} \sum ^\infty _{i_{NL}=0} \sum ^\infty _{j=0} \sum ^\infty _{k=0} p_{tb}^k H^*_{i_1, \dots , i_k, i_{NL}, j, k} \le 1\\ e^{-( A + C) I_M^*}&= {{\,\mathrm{\mathbbm {E}}\,}}\big [ p_{tb}^{N_I(t)} | N_A(t) + N_P(t) = 0 \big ] \cdot P(N_A(t) + N_P(t) = 0)\\&= \sum ^\infty _{i_1=0} \dots \sum ^\infty _{i_k=0} \sum ^\infty _{i_{NL}=0} \sum ^\infty _{k=0} p_{tb}^k H^*_{i_1, \dots , i_k, i_{NL}, 0, k} \le e^{-C I_M^*} \end{aligned}$$

from which we deduce that $A, C \ge 0$.

Recall from Sect. 3.2.1 that the existence of an endemic equilibrium solution is equivalent to the existence of a non-trivial solution to Eqs. (25) and (26). Characterising endemic equilibria is thus equivalent to characterising non-trivial points of intersection $I_M^* \in (0, P_M]$ of

$$\begin{aligned} F_1(I_M^*)&= \frac{g I_M^*}{\beta p_0 \big (\frac{P_M}{1 + g/\eta }- I_M^* \big )}\\ F_2(I_M^*)&= e^{-C I_M^*} - e^{-(A+C)I_M^*}. \end{aligned}$$

We note that $F_2(I_M^*) \ge 0$ for all $I_M^* \ge 0$ since $A, C \ge 0$, while $F_1(I^*_M) \ge 0$ only if $I_M^* \le \frac{P_M}{1 + g/\eta }$. We thus seek solutions in the domain $I_M^* \in (0, \frac{P_M}{1 + g/\eta } \big ]$.

Observe that $F_1(0) = F_2(0) = 0$, corresponding to the disease-free equilibrium. To characterise endemic equilibria, we compute the derivatives

$$\begin{aligned} F_1^{(n)}(I_M^*)&= n! \frac{g P_M}{\beta p_0 ( 1 + g/\eta )} \frac{1}{ \big ( \frac{P_M}{1 + g/\eta } - I_M^* \big )^{n+1}}\\ F_2^{(n)}(I_M^*)&= (-1)^n(C)^n e^{-C I_M^*} + (-1)^{n+1} (C+A)^n e^{-(C+A)I_M^*}. \end{aligned}$$

For all $I_M^* \in [0, \frac{P_M}{1 + g/\eta }]$, $F'_1(I_M^*), F_1''(I_M^*) >0$, so we conclude that $F_1$ is monotonically increasing and convex, with $F_1 \rightarrow \infty$ as $I_M^* \rightarrow \frac{P_M}{1 + g/\eta }$.

On the other hand, observing that $F_2'$, $F_2''$ are continuous; $F_2'(0) > 0$, $F_2^{''}(0) < 0$ and

$$\begin{aligned}&F_2'(M_0) = 0 \implies M_0 = \frac{1}{A} \log \Big (1 + \frac{A}{C} \Big )\\&F_2''(M_1) = 0 \implies M_1 = \frac{2}{A} \log \Big (1 + \frac{A}{C} \Big ) > M_0, \end{aligned}$$

by the intermediate value theorem, we deduce that $F_2$ is

- monotonically increasing, concave for all $I^*_M \in [0, M_0)$
- monotonically decreasing for all $I^*_M \in (M_0, \infty )$.

Setting $Q:= F_1' - F_2'$, we note that:

- $$Q'(I_M^*) > 0$$ for $$I_M^* \in [0, M_0)$$
- $$Q(I_M^*) > 0$$ for all $$I_M^* \in [M_0, \frac{P_M}{1+g/\eta }).$$

We thus consider two cases:

- **Case 1**: $$Q(0) > 0$$
  Then $Q(I_M^*) > 0$ for all $I_M^* \in [0, P_M] \implies (F_1 - F_2)$ is monotonically increasing for all $I_M^* \in [0, 1]$. Since $(F_1-F_2)(0) = 0$, there exists no non-trivial point of intersection.
- **Case 2**: $$Q(0) < 0$$
  Since *Q* is monotonically increasing on the interval $$[0, M_0)$$ with $$Q(0)< 0 < Q(M_0)$$, by the intermediate value theorem, there exists $$M_1 \in (0, M_0)$$ such that $$Q(I_M^*) \le 0$$ for $$I_M^* \in [0, M_1]$$ and $$Q(I_M^*) > 0$$ for $$I_M^* \in (M_1, \infty )$$. Noting that $$(F_1 - F_2)(0) = 0$$, it follows that $$(F_1 - F_2)$$ is montonically decreasing and negative on the interval $$(0, M_1)$$; but monotonically increasing on the interval $$(M_1, \frac{P_M}{1+g/\eta })$$. Since $$(F_1 - F_2)(I_M^*) \rightarrow + \infty $$ as $$I_M^* \rightarrow \frac{P_M}{1 + g/\eta }$$, by the intermediate value theorem, there exists a unique non-trivial root of $$(F_1 - F_2)$$ in the interval $$(M_1, \frac{P_M}{1 + g/\eta })$$.

Therefore, $F_1$ and $F_2$ have at most one non-zero point of intersection if and only if

$$\begin{aligned} Q(0) < 0 \iff R_0^2 := \frac{A \cdot \beta p_0 P_M}{g (1 + g/\eta )} > 1. \end{aligned}$$

As such, $R_0 > 1$ is a sufficient and necessary condition for the existence of an endemic equilibrium; when $R_0 > 1$, the endemic equilibrium solution is unique.

### Interpretation of the quantity $R_0$

Here, we provide an explicit interpretation of the integral term in the expression for $R_0$ (Eq. (27)). Following the argument detailed in Mehra et al. (2022), given a single infective bite at time zero, the PGF for the occupancy $M_s(\tau )$ of queue $s \in S$ at time $\tau > 0$ can be written

$$\begin{aligned} {{\,\mathrm{\mathbbm {E}}\,}}\bigg [ \prod _{s \in S} z_s^{M_s(\tau )} \bigg ] = \frac{\sum _{s \in S_p} z_s \cdot p_{p, s}(\tau )}{1 + \nu \big ( 1 - \sum _{s \in S_h} z_s \cdot p_{h, s}(\tau )\big )}. \end{aligned}$$

Suppose a mosquito takes a bloodmeal at time $\tau$. The probability of successful human-to-mosquito transmission—adjusting for the acquisition of transmission-blocking immunity attributable to the bite—at time $\tau$ can be written

$$\begin{aligned} p_{h \rightarrow m}(t)&= p_0 \Big ( {{\,\mathrm{\mathbbm {E}}\,}}\Big [ p_{tb}^{M_I(t)} \Big ] - {{\,\mathrm{\mathbbm {E}}\,}}\Big [ p_{tb}^{M_I(t)} \big | M_A(t) + M_P(t) = 0 \Big ] \cdot P \big ( M_A(t) + M_P(t) = 0 \big ) \Big )\\&= p_0 \bigg ( \frac{1 - (1-p_{tb}) p_{p, I}(\tau )}{1 + \nu (1-p_{tb}) p_{h, I}(\tau )} - \frac{1 - (1-p_{tb}) \cdot p_{p, I}(\tau ) - p_{p, A}(\tau ) }{1 + \nu (1-p_{tb}) p_{h, I}(\tau ) + \nu p_{h, A}(\tau )} \bigg ) \end{aligned}$$

in direct analogy to the Eq. (16). The total expected duration $T_{h \rightarrow m}$ of *transmissible* blood-stage infection attributable to the bite can therefore be written

$$\begin{aligned} T_{h \rightarrow m} = \int ^\infty _{0} p_{h \rightarrow m}(\tau ) d \tau , \end{aligned}$$

which is precisely the integral term in Eq. (27).

## References

[CR1] Almeida ACG (2018). High proportions of asymptomatic and submicroscopic Plasmodium vivax infections in a peri-urban area of low transmission in the Brazilian Amazon. Parasites Vectors.

[CR2] Antonelli LR, Junqueira C, Vinetz JM, Golenbock DT, Ferreira MU, Gazzinelli RT (2020). The immunology of Plasmodium vivax malaria. Immunol Rev.

[CR3] Anwar MN, Hickson RI, Mehra S, MMcCaw J, Flegg JA (2022). A multiscale mathematical model of plasmodium Vivax transmission. Bull Math Biol.

[CR4] Barbour AD, Luczak MJ (2012). A law of large numbers approximation for Markov population processes with countably many types. Probab Theory Relat Fields.

[CR5] Battle KE (2014). Geographical variation in Plasmodium vivax relapse. Malar J.

[CR6] Battle KE (2019). Mapping the global endemicity and clinical burden of Plasmodium vivax, 2000–17: a spatial and temporal modelling study. The Lancet.

[CR7] Battle KE, Kevin Baird J (2021). The global burden of Plasmodium vivax malaria is obscure and insidious. PLoS Med.

[CR8] Brauer F (1978). Asymptotic stability of a class of integro-differential equations. J Differ Equ.

[CR9] Commons RJ (2019). Risk of Plasmodium vivax parasitaemia after Plasmodium falciparum infection: a systematic review and meta-analysis. Lancet Infect Dis.

[CR10] de Jong RM, Tebeje SK, Meerstein-Kessel L, Tadesse FG, Jore MM, Stone W, Bousema T (2020). Immunity against sexual stage Plasmodium falciparum and Plasmodium vivax parasites. Immunol Rev.

[CR11] De Roode JC (2005). Virulence and competitive ability in genetically diverse malaria infections. Proc Natl Acad Sci.

[CR12] De Zoysa AP, Mendis C, Gamage-Mendis AC, Weerasinghe S, Herath PR, Mendis KN (1991). A mathematical model for Plasmodium vivax malaria transmission: estimation of the impact of transmission-blocking immunity in an endemic area. Bull World Health Organ.

[CR13] Dennis Shanks G, White NJ (2013). The activation of vivax malaria hypnozoites by infectious diseases. Lancet Infect Dis.

[CR14] Deroost K, Pham T-T, Opdenakker G, Van den Steen PE (2016). The immunological balance between host and parasite in malaria. FEMS Microbiol Rev.

[CR15] Diekmann O, Heesterbeek JAP, Metz JAJ (1990). On the definition and the computation of the basic reproduction ratio R0 in models for infectious diseases in heterogeneous populations. J Math Biol.

[CR16] Feller W (1968). An introduction to probability theory and its applications.

[CR17] Ferreira MU (2022). Relative contribution of low-density and asymptomatic infections to Plasmodium vivax transmission in the Amazon: pooled analysis of individual participant data from population-based cross-sectional surveys. Lancet Region Health-Am.

[CR18] Galinski MR, Barnwell JW (2008). Plasmodium vivax: Who cares?. Malar J.

[CR19] Gamage-Mendis AC, Rajakaruna J, Carter R, Mendis KN (1992). Transmission blocking immunity to human Plasmodium vivax malaria in an endemic population in Kataragama, Sri Lanka. Parasite Immunol.

[CR20] Garrett-Jones C (1964). The human blood index of malaria vectors in relation to epidemiological assessment. Bull World Health Organ.

[CR21] Gething PW, Van Boeckel TP, Smith DL, Guerra CA, Patil AP, Snow RW, Hay SI (2011). Modelling the global constraints of temperature on transmission of Plasmodium falciparum and P. vivax. Parasites Vectors.

[CR22] Griffin JT (2010). Reducing Plasmodium falciparum malaria transmission in Africa: a model-based evaluation of intervention strategies. PLoS Med.

[CR23] Harrison JM, Lemoine AJ (1981). A note on networks of infinite-server queues. J Appl Probab.

[CR24] Henry JM (2020). A hybrid model for the effects of treatment and demography on malaria superinfection. J Theor Biol.

[CR25] Ishikawa H, Ishii A, Nagai N, Ohmae H, Harada M, Suguri S, Leafasia J (2003). A mathematical model for the transmission of Plasmodium vivax malaria. Parasitol Int.

[CR26] Jeffrey A, Zwillinger D (2007). Table of integrals, series, and products.

[CR27] Joyner CJ (2019). Humoral immunity prevents clinical malaria during Plasmodium relapses without eliminating gametocytes. PLoS Pathog.

[CR28] Kammanee A, Kanyamee N, Tang IM (2001). Basic reproduction number for the transmission of Plasmodium vivax malaria. Southeast Asian J Trop Med Public Health.

[CR29] Koepfli C, Colborn KL, Kiniboro B, Lin E, Speed TP, Siba PM, Felger I, Mueller I (2013). A high force of Plasmodium vivax blood-stage infection drives the rapid acquisition of immunity in Papua New Guinean children. PLoS Negl Trop Dis.

[CR30] Le Menach A, Shannon Takala F, McKenzie E, Perisse A, Harris A, Flahault A, Smith DL (2007). An elaborated feeding cycle model for reductions in vectorial capacity of night-biting mosquitoes by insecticide-treated nets. Malar J.

[CR31] Mehra S, McCaw JM, Flegg MB, Taylor PG, Flegg JA (2020). An Activation-Clearance Model for Plasmodium vivax Malaria. Bull Math Biol.

[CR32] Mehra S, McCaw JM, Flegg MB, Taylor PG, Flegg JA (2021). Antibody dynamics for Plasmodium vivax Malaria: a mathematical model. Bull Math Biol.

[CR33] Mehra S, Stadler E, Khoury D, McCaw JM, Flegg JA (2022). Hypnozoite dynamics for Plasmodium vivax malaria: the epidemiological effects of radical cure. J Theor Biol.

[CR34] Mehra S, McCaw JM, Taylor PG (2023) Superinfection and the hypnozoite reservoir for Plasmodium vivax: a general framework. J Math Biol. 10.1007/s00285-023-02014-3 PMC10692056 38040981

[CR35] Mueller I, Galinski MR, Tsuboi T, Arevalo-Herrera M, Collins WE, King CL (2013). Natural acquisition of immunity to Plasmodium vivax: epidemiological observations and potential targets. Adv Parasitol.

[CR36] Mueller I, Shakri AR, Chitnis CE (2015). Development of vaccines for Plasmodium vivax malaria. Vaccine.

[CR37] Nåsell I (2013). Hybrid models of tropical infections.

[CR38] Popovici J (2018). Genomic analyses reveal the common occurrence and complexity of Plasmodium vivax relapses in Cambodia. MBio.

[CR39] Ricardo A, Ferreira Marcelo U, Gabriela M, Gomes M (2012). Modeling the effects of relapse in the transmission dynamics of malaria parasites. J Parasitol Res.

[CR40] Roy M, Bouma MJ, Ionides EL, Dhiman RC, Pascual M (2013). The potential elimination of Plasmodium vivax malaria by relapse treatment: insights from a transmission model and surveillance data from NW India. PLoS Negl Trop Dis.

[CR41] Tadesse FG (2018). The relative contribution of symptomatic and asymptomatic Plasmodium vivax and Plasmodium falciparum infections to the infectious reservoir in a low-endemic setting in Ethiopia. Clin Infect Dis.

[CR42] White, Michael T, Karl S, Battle KE, Hay SI, Mueller I, Ghani AC (2014) Modelling the contribution of the hypnozoite reservoir to *Plasmodium vivax* transmission. eLife 3:e04692 10.7554/eLife.04692 PMC4270097 25406065

[CR43] White NJ (2016). Why do some primate malarias relapse?. Trends Parasitol.

[CR44] White NJ (2017). Malaria parasite clearance. Malar J.

[CR45] White MT (2018). Mathematical modelling of the impact of expanding levels of malaria control interventions on Plasmodium vivax. Nat Commun.

[CR46] White MT (2018). Plasmodium vivax and Plasmodium falciparum infection dynamics: re-infections, recrudescences and relapses. Malar J.

[CR47] White MT, Griffin JT, Akpogheneta O, Conway DJ, Koram KA, Riley EM, Ghani AC (2014). Dynamics of the antibody response to Plasmodium falciparum infection in African children. J Infect Dis.

[CR48] White MT, Shirreff G, Karl S, Ghani AC, Mueller I (2016). Variation in relapse frequency and the transmission potential of Plasmodium vivax malaria. Proc R Soc B: Biol Sci.

[CR49] White M, Amino R, Mueller I (2017). Theoretical implications of a pre-erythrocytic Plasmodium vivax vaccine for preventing relapses. Trends Parasitol.

[CR50] WHO (2021) World Malaria Report 2021

[CR51] Xekalaki E (1987). A method for obtaining the probability distribution of m components conditional on $$\ell $$ components of a random sample. Rev Roumaine Math Pure Appl.

